# Impaired Reliability and Precision of Spiking in Adults But Not Juveniles in a Mouse Model of Fragile X Syndrome

**DOI:** 10.1523/ENEURO.0217-19.2019

**Published:** 2019-12-02

**Authors:** Deepanjali Dwivedi, Sumantra Chattarji, Upinder S. Bhalla

**Affiliations:** 1 National Centre for Biological Sciences, TIFR, Bellary Road, Bangalore 560065, India; 2 Centre for Brain Development and Repair, Institute for Stem Cell Biology and Regenerative Medicine, Bangalore 560065, India; 3 Centre for Discovery Brain Sciences, Deanery of Biomedical Sciences, University of Edinburgh, EH8 9XD Edinburgh, United Kingdom

**Keywords:** fragile X syndrome, ion channels, neurodeveloment, SK channels

## Abstract

Fragile X syndrome (FXS) is the most common source of intellectual disability and autism. Extensive studies have been performed on the network and behavioral correlates of the syndrome, but our knowledge about intrinsic conductance changes is still limited. In this study, we show a differential effect of FMRP knockout in different subsections of hippocampus using whole-cell patch clamp in mouse hippocampal slices. We observed no significant change in spike numbers in the CA1 region of hippocampus, but a significant increase in CA3, in juvenile mice. However, in adult mice we see a reduction in spike number in the CA1 with no significant difference in CA3. In addition, we see increased variability in spike numbers in CA1 cells following a variety of steady and modulated current step protocols. This effect emerges in adult mice (8 weeks) but not juvenile mice (4 weeks). This increased spiking variability was correlated with reduced spike number and with elevated AHP. The increased AHP arose from elevated SK currents (small conductance calcium-activated potassium channels), but other currents involved in medium AHP, such as *I*_h_ and M, were not significantly different. We obtained a partial rescue of the cellular variability phenotype when we blocked SK current using the specific blocker apamin. Our observations provide a single-cell correlate of the network observations of response variability and loss of synchronization, and suggest that the elevation of SK currents in FXS may provide a partial mechanistic explanation for this difference.

## Visual Abstract

**Figure:**
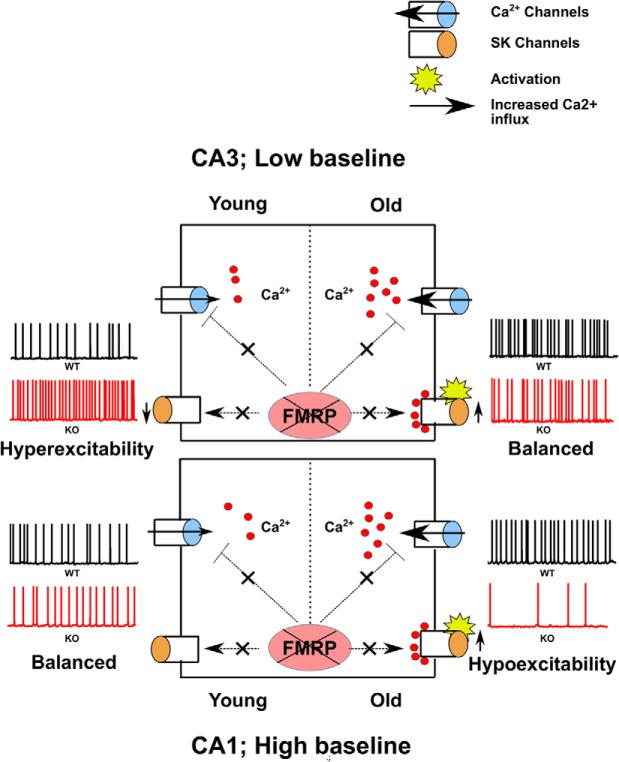

## Significance Statement

Fragile X syndrome leads to a range of intellectual disability effects and autism. We have found a differential effect of FMRP KO in different subsections of hippocampus where it caused an increased spiking in CA3 in juveniles and reduced spiking in CA1, in adults. We have also found that even individual neurons with this mutation exhibit increased variability in their activity patterns. Importantly, this effect emerges after 6 weeks of age in mice. We showed that a specific ion channel protein, the SK channel, was partially responsible, and blockage of these channels led to a partial restoration of cellular activity. This is interesting as it provides a possible molecular link between activity variability in single cells and reported irregularity in network activity.

## Introduction

Fragile X syndrome (FXS) is an autism spectrum disorder arising from increased repeats of CGG trinucleotide in the *FMR1* gene. This leads to silencing of the gene and hence to the absence of the FMRP protein (O’Donnell and Warren, 2002). FMRP is a transcription factor that affects multiple downstream genes including ion channels (Brown et al., 2001; Darnell et al., 2011). Studies have shown that the absence of FMRP affects the functioning of ion channels either by regulating the number of channels (Meredith et al., 2007; Strumbos et al., 2010; Gross et al., 2011; Lee et al., 2011a; Routh et al., 2013; Ferron et al., 2014; Zhu et al., 2018) or by directly interacting with the channels (Brown et al., 2010; Deng et al., 2013). Multiple studies have shown that potassium channels have a significant effect on spike precision (Fricker and Miles, 2000; Gittelman, 2006; Cudmore et al., 2010), and many of these potassium channels are transcription hits for FMRP. This leads to the hypothesis that FXS may alter the functioning of one or multiple potassium channels, leading to effects on spike precision.

In recent years a number of *in vivo* hippocampal recording studies have shown that there is poor correlation of spiking activity between cells, and abnormal theta phase–gamma phase coupling in FXS mice (Radwan et al., 2016; Arbab et al., 2018a,b; Talbot et al., 2018). In medial prefrontal cortex, variability in calcium (Ca^2+^) responses has also been observed, leading to impaired spike timing-dependent plasticity (STDP) (Meredith et al., 2007).These studies have led to the “discoordination hypothesis” for FXS (Talbot et al., 2018). This hypothesis states that neurons in FXS are uncorrelated and have aberrant network discharges. In apparent contradiction to this hypothesis, neurons showed hyperconnectivity and synchronization in cortical networks of FXS model mice (Testa-Silva et al., 2012; Gonçalves et al., 2013).

Synchronicity is an emergent property of a network and is a function of both network connectivity and intrinsic properties. Specifically, potassium conductance has been shown to have significant effects on spike precision and network synchrony (Fricker and Miles, 2000; Pfeuty et al., 2003; Deister et al., 2009; Cudmore et al., 2010; Gastrein et al., 2011; Hou et al., 2012). Modeling studies have also shown that conductance that mediates spike frequency adaptation helps to synchronize network firing (Crook et al., 1998). *I*_h_ currents, *I*_M_ currents, and SK currents have been shown to mediate adaptation for phase precession in hippocampal neurons, (Orbán et al., 2006; Kwag et al., 2014) spiking reliability in insect neurons (Gabbiani and Dewell, 2018) and also to maintain regular firing in robust nucleus of acropallium in zebra finches (Hou et al., 2012). Thus FXS mutations, through their effects on potassium channels, may lead to several of the observed network dysfunctions in FXS models.

In the present study, we tested alterations in intrinsic properties of the cell that might underlie the above network observations of uncorrelated activity. First, we observed that cells from FXS knock-out (KO) hippocampal slices were more unreliable, both at within-cell and between-cell levels, than controls. Consistent with the developmental manifestations of FXS, this effect was absent in juvenile mice and emerged only after ∼6 weeks. Decreased spiking in KO mice was correlated with increased variability. At the mechanistic level, we found that the spiking changes were due to elevated medium afterhyperpolarization (mAHP), which in turn was due to elevated SK currents. In contrast to these observations of decreased spiking in the CA1 region of adult animals, we observed increased spiking in the CA3 region of juvenile animals (Deng et al., 2019), but not in the CA1 region. Consistent with earlier observations (Deng et al., 2019), we did not see any significant change in the spatial distribution of SK channels in either CA3 or CA1. Finally, we tested the outcome of the blockers of SK and found that that they indeed led to a partial rescue of the observed phenotype of increased spiking variability and reduced timing precision in adult animals.

## Materials and Methods

### Animals

C57BL/6 strain of *Fmr1tmCgr* male mice were used for the experiments. All experimental procedures were approved by the National Centre for Biological Sciences ethics committee [Project ID: NCBS-IAE-2017/04(N)]. The animals were housed in the institute animal house where they were maintained on a 12 h light/dark cycle. The animals used were from an older animal group in the range of 6–8 weeks of age; the younger group was 3–4 weeks of age.

### Slice preparation

Mice were anesthetized with halothane. Their head was decapitated after they were killed by cervical dislocation. Hippocampal slices were made in the ice-cold aCSF of the following composition: 115 mm NaCl, 25 mm glucose, 25.5 mm NaHCO_3_, 1.05 mm NaH_2_PO_4_, 3.3 mm KCl, 2 mm CaCl_2_, and 1 mm MgCl_2_. 400-μm-thick slices were made using a VT1200S vibratome and then incubated at room temperature for 1 h in the aCSF, which was constantly bubbled with 95% O_2_ and 5% CO_2_. Subsequently, the slices were transferred to the recording chamber where they were maintained at an elevated temperature of 30–34^°^C for the recordings.

### Electrophysiology

CA1 neurons were identified under an upright differential interference contrast microscope (BX1WI microscope, Olympus) using a 40× objective (water immersion lens, 0.9 numerical aperture, LUMPLFLN, 40×). 2–4 MΩ pipettes were pulled from thick-walled borosilicate glass capillaries on a P-1000 Flaming Micropipette Puller (Sutter Instrument). The pipettes were filled with internal solution of the following composition for whole-cell current-clamp recordings: 120 mm potassium gluconate, 20 mm KCl, 0.2 mm EGTA, 4 mm NaCl,10 mm HEPES buffer, 10 mm phosphocreatine, 4 mm Mg-ATP, and 0.3 mm Na-GTP, at (pH 7.4 and 295 mOsm). For voltage-clamp recordings, the same composition of internal solution was used with the one change: 120 mm potassium gluconate was substituted with 120 mm potassium methylsulphate. Cells were recorded if they had a resting potential of ≤60 mV. We also required that they exhibit stable firing with little or no depolarization block for lower current inputs. Series resistance and input resistance were continuously monitored during the protocols, and the cell was discarded if these parameters changed by >25%.

### Protocol for measuring spike variability and analysis

All spike variability and precision experiments were performed in current-clamp mode. A step input current stimulus of 150 pA DC for 900 ms was used for the majority of the recordings. In some cases, as indicated in the text, frozen noise and sinusoidal input currents were also used, riding on a baseline current step of 150 pA, and again for a duration of 900 ms. The SDs of noise used in noise protocols were 10, 25, 50, and 100 pA with a time cutoff of τ = 3 ms. For sinusoidal currents, SDs of 50 and 100 pA were used at 5 Hz. All the protocols were repeated for 25 trials. Within-cell spike variability (CV*_w_*) was computed as coefficient of variation (CV) in spike numbers across 25 trials. Spike numbers computed for all 25 trials were used to find the SD in spike numbers, which was then divided by the mean number of spikes to find CV*_w_*, as follows:

(1) $${\left({\text{CV}}_{\text{w}}\right)}_{\text{n}}=\sigma_{\text{n}}/\mu_{\text{n}},$$

where *n* is the cell index, σ*_n_* is the SD across trials within cell *n*, and μ*_n_* is the mean number of spikes across all the trials within cell *n*.

**Figure 1.**
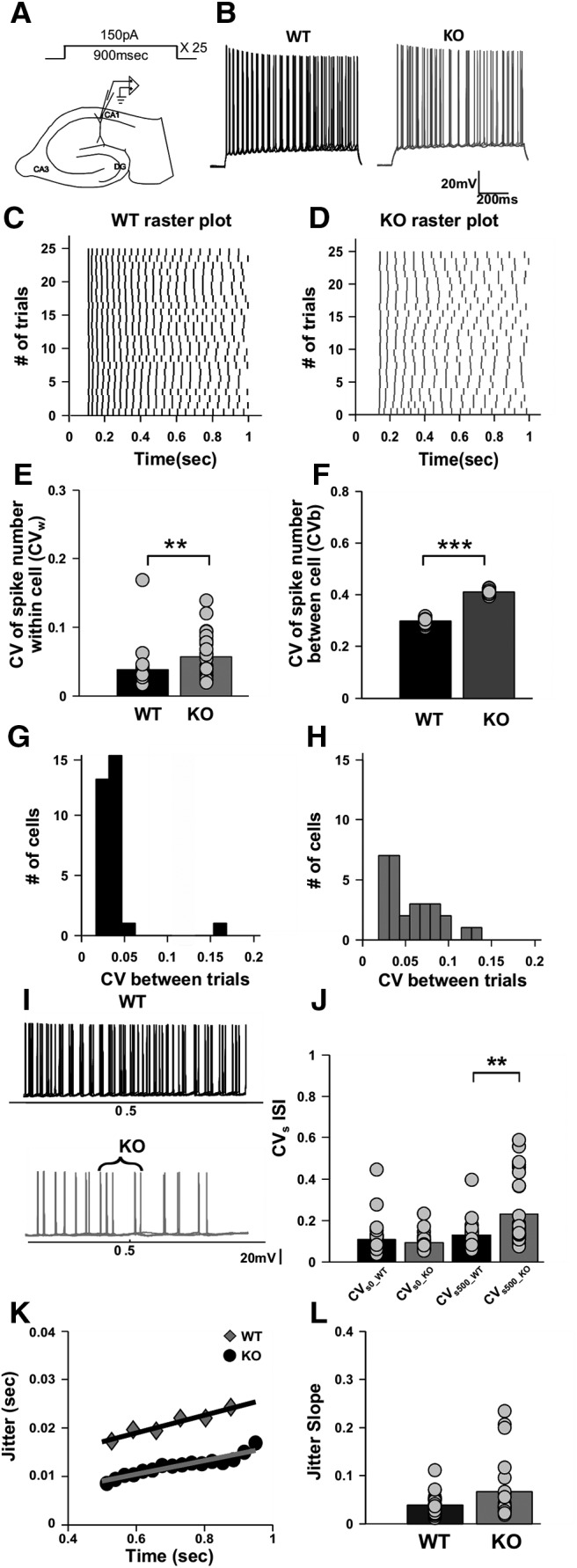
Increased spike variability and spike timing imprecision both at the within-cell level (Co-efficient of Variation - CV*_w_*) across multiple trials and between cells (Co-efficient of Variation - CV*_b_*) in CA1 hippocampus of FXS animals. ***A***, Schematic of a hippocampal slice and the step depolarization protocol. The protocol consists of a 150 pA step for 900 ms time duration repeated over 25 trials. ***B***, Representative raw traces of spiking, superimposed for four trials only for WT (black) and KO (gray). Calibration: 20 mV, 200 ms. ***C***, ***D***, Representative raster plot showing spiking for the step depolarization protocol for WT (black; ***C***) and KO (gray; ***D***). ***E***, Bar plot for within-cell variability across multiple trials (CV*_w_*) for WT (*n* = 30 cells, black) and KO (*n* = 29 cells, gray). KO cells have significantly higher within-cell variability than WT cells (*p* = 0.0048, Wilcoxon rank sum test). ***F***, Bar plot for between-cell variability (CV*_b_*) for matched trials, WT (*n* = 30 cells, black) and KO (*n* = 29 cells, gray). KO cells have significantly higher between cell variability than WT cells (*p* < 0.001, Wilcoxon rank sum test). ***G***, ***H***, Histogram showing the spread of the CV*_w_* parameter for WT (*n* = 30 cells, black; ***G***) and for KO (*n* = 29 cells, gray; ***H***). KO cells have more spread in CV*_w_* parameter, depicting heterogeneity in KO cell population. ***I***, Representative trace showing the time window where CV_s_ in ISI is calculated for WT (top, black trace) and for KO (bottom, gray trace). Superimposed traces are for four trials only. A curly bracket in the KO trace shows the time window in which CV*_s_*_500_ is calculated. Calibration: 20 mV. ***J***, Bar plot for CV*_s_* in ISI, a measure of spike precision for WT (*n* = 30 cells, black) and KO (*n* = 29 cells, gray). CV*_s_*_0_ is not significantly different between WT and KO. However, there is a significant increase in the CV*_s_*_500_ for KO cells compared with WT (*p* = 0.018 for WT vs KO differences; two-way ANOVA). ***K***, Representative trace showing jitter in spike timings for one WT cell (black circles) and one KO cell (gray diamonds). The straight line is a regression fit to the points, and its slope is a measure of the increase in jitter with time. Jitter in spike timings are calculated only for the last 400 ms of the trial. ***L***, Bar plot for jitter slope, a measure of spike precision for WT (*n* = 29 cells, black) and KO (*n* = 23 cells, gray). The jitter slope is not significantly different between WT and KO cells (*p* = 0.14; Wilcoxon rank sum test). ***p < 0.001, **p < 0.01.

The array of floating point numbers (i.e., vector for CV*_w_*) was computed for all recorded KO and wild-type (WT) cells, respectively, and was used for comparing the responses (Fig. 1*E*). The distributions in CV*_w_* were compared using the Wilcoxon test.

The analysis of between-cell spiking variability (CV*_b_*) was derived from an article that reports experiments on network synchrony (Talbot et al., 2018). In contrast to our CV*_w_* analysis, where variability in spiking was assessed within each cell, CV*_b_* measures the variability across trials between cells. For computing CV*_b_*, spike numbers on corresponding trial numbers were compared between different cells. Specifically, we constructed a vector for each cell whose entries were the number of spikes for successive trials. These vectors were compared across different cells within the same genotype and were used to find CV*_b_*. The following formula was used for the computation:

(2) $${\left({\text{CV}}_{\text{b}}\right)}_{\text{i}}=\sigma_{\text{i}}/\mu_{\text{i}},$$

where *i* is the trial index, σ_i_ is the SD over trial *i* between cells, and μ*_i_*is the mean number of spikes on trial *i* between all cells. The CV*_b_* vectors were computed for WT and KO populations, respectively (Fig. 1*F*), and compared using Wilcoxon statistics.

### Measurement of spike timing precision

#### Measurement of spike timing precision: CV_s_

Spike times were calculated for all the spikes in all trials. The interspike interval (ISI) was computed for the first two spikes and for spikes that bracketed a time of 500 ms after the start of the step input protocol (Fig. 1*I*). For each cell *m*, we obtained the coefficient of variation, CV*_s_*_0__*_m_* and CV*_s_*_500__*_m_* for the first ISI and the ISI at 500 ms, respectively, as follows:

(3) $${\text{CV}}_{\text{s}0}{\_}_{\text{m}}=\sigma_{0\text{m}}/\mu_{0\text{m}},$$

where *m* is the cell index, σ*_m_* is the SD of ISI across trials within cell *m*, and μ*_m_* is the mean ISI across all the trials for cell *m*. We calculated CV*_s_*_500__*_m_* in a similar manner.

By repeating this calculation over all cells, we obtained the following four vectors: CV_s0_WT_, CV*_s_*_500_WT_, CV_s0_KO_, and CV*_s_*_500_KO_. The distributions in these four vectors were compared using a two-way ANOVA (Fig. 1*J*).

#### Jitter slope

The jitter slope analysis was taken from the study by Bacci and Huguenard (2006). Jitter was defined as σ*_ti_*, which is the SD of spike timing for spike number *i*, compared over all trials for a given cell. In this manner, the vector σ*_t_* was obtained by considering all the spike numbers. Note that not all trials had the same number of spikes, so the final few spike numbers may have had fewer samples in σ*_t_*.

The jitter slope was obtained as a regression fit for the vector σ*_t_*, with the *x*-axis defined by the corresponding vector for mean timings μ*_t_*. This slope was calculated for spikes falling in the last 400 ms of the current pulse since this is where the SK channel is activated. As a *pre hoc* criterion, cells were required to have a minimum of two spike time points to be selected for this calculation. Note that this criterion excluded several cells that had strong suppression of firing toward the end of the trial, presumably due to SK channel activation. Thus, this measure was compromised by being insensitive to precisely those cells with the largest effects in the KO.

### Measurement of frequency versus current curves

The measurement of frequency versus current (f–I) curves was performed in current clamp. For plotting f–I curves, each cell was given input currents from −100 to 400 pA at its resting membrane potential (RMP). The mean ± SEM of the number of spikes elicited for each input current was plotted on the *y*-axis, and the corresponding input current on the *x*-axis.

### AHP measurement

AHPs were measured by holding the cell at −70 mV in current-clamp mode, using some holding current. In each trial, the holding current was set to 0 for 200 ms, and then step input for 100 ms was delivered. The current during this step input was adjusted so that the cell produced five spikes in 100 ms. This stimulus was repeated for 15 trials, and the voltage traces of all the trials were averaged. AHPs were analyzed as the hyperpolarization after the spike train. mAHP was computed as the average hyperpolarization 50 ms after the spike train, and slow AHP (sAHP) was computed 200 ms after the spike train (see Fig. 5*A*,*B*).

### Immunofluorescence experiments and analysis

The 3- to 4-week-old or 6- to 8-week-old animals were anesthetized, and brains were dissected out. Brains were fixed in 4% PFA for 48 h at 4°C. Postfixation, brains were washed with PBS, and 30-μm-thick slices were cut on a VT12000S vibratome. Slices were kept in cryoprotectant solution and stored at −20°C for further use. Slices were resuspended in PBS solution for the experiment. 50 mM ammonium chloride (NH_4_Cl) was used to quench autofluorescence from the samples. After multiple steps of washing and permeabilization in 0.3% PBS with Triton X-100, cells were incubated with 5% normal goat serum (NGS) for blocking for 1–2 h. Followed by washes, slices were incubated in primary antibody for the SK2 channel, overnight at 4°C. Primary antibody was purchased from Alomone Labs (catalog #APC–028) and were used in 1:300 dilution with 2.5% NGS. Further, slices were incubated in secondary antibody at 1:500 dilution for 1–2 h at room temperature. After several washes and DAPI staining (Sigma-Aldrich) for 15 min in PBS, slices were mounted in Prolong Gold Mounting Media and imaged with an Olympus FV3000 confocal microscope.

### Measurement of M and *I*_h_ currents

For measuring the *I*_h_ sag produced in voltage change, cells were held at −70 mV and given a step hyperpolarization of 250 pA for 500 ms in current-clamp mode. Voltage deflection was calculated by finding the percentage change between maximum and steady-state voltages during the hyperpolarizing pulse [(*V*_max_ − *V*_ss_)/*V*_max_] * 100. Each cell was presented with 15 trials, and *I*_h_ sag was averaged across all the trials to get a value for a cell.

TTX (0.5 μm) was added in the bath while measuring M currents in voltage clamp. For measuring M currents, cells were held at −20 mV, and M currents were deactivated in steps from −20 to −80 mV with a difference of 10 mV in a 900 ms time duration. The difference between the steady-state current after the start of the protocol (from the last 250 ms) and the baseline of the cell was quantified as the current elicited due to the protocol. Specific M current blocker XE991 (30 μm) was perfused to get the postblocker trace. The difference between pre- and post-XE991 perfusion was quantified as the M current.

### Measurement of SK currents

TTX (0.5 μm) was added into the bath while measuring SK currents in voltage clamp. For measuring SK currents, cells were held at −55 mV while increasing voltage steps were given from −55 to +35 mV, in increments of 10 mV, for 800 ms duration. Each voltage step was preceded with a small hyperpolarization from −55 to −65 mV for 100 ms. Cell capacitance compensation was performed during the recordings. The SK currents were estimated from the amplitude of the outward current 25 ms after the voltage was returned to the holding potential as the initial part of the trace will be contaminated with capacitance currents (see Fig. 6*A*,*B*). Apamin perfusion (100 nm) was performed to identify these currents. The same protocol was used post-apamin perfusion. The difference in current between pre-apamin and post-apamin perfusion was quantified to find the apamin-sensitive SK current.

### Data acquisition and analysis

Recordings were performed using an Axopatch 200 B Amplifier and digitized using a Digidata 1400A digitizer. Current-clamp recordings were filtered at 10 kHz and sampled at 20 kHz. Voltage-clamp recordings were conducted at a gain of 5, filtered at 3 kHz, and sampled at 20 kHz. All analyses were performed using custom-written code in MATLAB (R2013a) and GraphPad Prism 6. Statistical tests were performed after checking the normality of the data. We plotted the data to check for normality. Even if the data looked normal, a Wilcoxon rank sum test was used for most analysis for more stringent results. For multiple comparisons, ANOVA was used with Sidak’s *post hoc* test or *t* test with Bonferroni correction as *post hoc* tests. All data are plotted as the mean ± SEM.

### Drugs and chemicals used

All toxins were purchased from Sigma-Aldrich except for TTX, which was from Hello Bio. TTX (stock solution of 0.5 mm) and apamin (stock solution of 1 mm) were made with Milli-Q water. XE991 (stock solution of 10 mm) was made in DMSO. All stocks were stored at −20^°^C and were diluted to the working concentration immediately before the experiments.

### Experiment design and statistical analysis

We have used hippocampal slice preparation from male mice in this study, where CA1 and CA3 regions were used for electrical recordings. Different current inputs were injected into the cell soma via recording electrode, and elicited spikes were recorded in current-clamp mode. Spiking variability was assessed by finding the CV for within a cell and between cells. Spike precision was assessed by comparing CV ISI occurring in the initial part of the trail with the ISI occurring around 500ms post commencement of the trial. SK currents were measured in voltage-clamp mode and were found to be partially responsible for increased variability. In addition, we found a differential effect of FMRP KO in different subsections of hippocampus. In young animals, we observed KOs having hyperexcitability for CA3 cells with no difference in CA1 cells. In older animals, we saw that the hyperexcitability seen in CA3 goes away while there is an emergence of hypoexcitability in CA1 cells. All analysis and plotting was performed in MATLAB and GraphPad 6. The normality of the data was checked by plotting it. Even if the distribution followed a normal distribution profile, a Wilcoxon test was used. For multiple comparisons, two-way or three-way ANOVA with Sidak’s *post hoc* test or *t* test using a Bonferroni correction were used as the *post hoc* test.

## Results

### FMR1 KO cells exhibit increased variability in spike numbers and impaired spike precision

**Figure 2.**
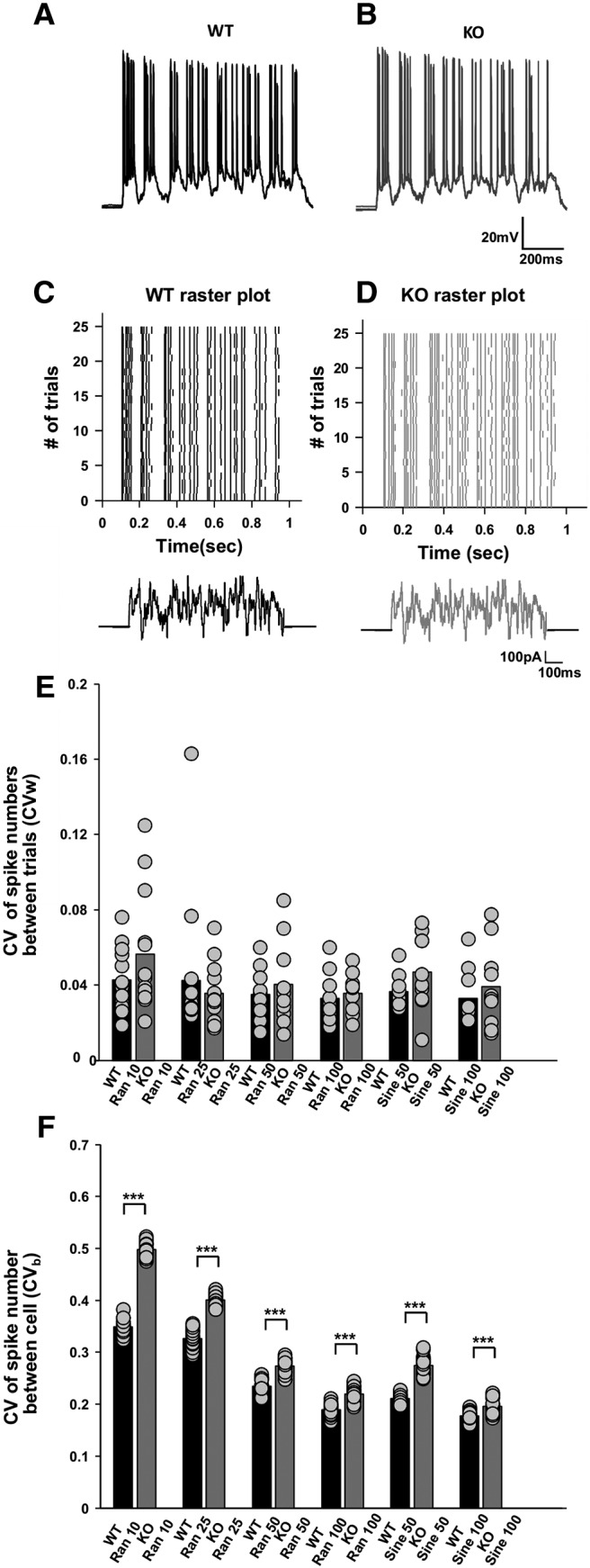
KO cells have increased CV*_b_* for noise and sinusoidal stimuli but not CV*_w_* across trials. ***A***, ***B***, Representative raw plot of spiking showing no spike variability or spike precision differences for the Random 100 noise protocol across multiple trials for WT (black; ***A***) and for KO (gray; ***B***). The trace is for four superimposed trials. Calibration: 20 mV, 200 ms. ***C***, ***D***, Raster plot showing no spike variability or spike precision differences for Random 100 noise protocol across multiple trials for WT (black; ***C***) and for KO (gray; ***D***). Trace below indicates Random 100 input protocol. Calibration: 100 pA, 100 ms. ***E***, Bar plot for within-cell variability differences (CV*_w_*) for all noise and sinusoidal protocols for WT (black bars) and KO (gray bars). The protocol is numbered by the SD in pA (random protocols) or sine-wave amplitude (sine protocols; Random 10 protocol: *n* = 16 for WT and *n* = 13 for KO; Random 25 protocol: *n* = 16 for WT and *n* = 13 for KO; Random 50 protocol: *n* = 10 for both WT and KO; Random 100 protocol: *n* = 10 for both WT and KO; Sine 50 protocol: *n* = 10 for both WT and KO; Sine 100 protocol: *n* = 10 for both WT and KO; circles represent individual data points). No significant within-cell variability (CV*_w_*) differences were seen between WT and KO cells in any protocol. ***F***, Bar plot for between-cell variability differences (CV*_b_*) for all noise and sinusoidal protocols (Random 10 protocol: *n* = 16 for WT and *n* = 13 for KO; Random 25 protocol: *n* = 16 for WT and *n* = 13 for KO; Random 50 protocol: *n* = 10 for both WT and KO; Random 100 protocol: *n* = 10 for both WT and KO; Sine 50 protocol: *n* = 10 for both WT and KO; Sine 100 protocol: *n* = 10 for both WT and KO). Significant differences were seen for all pairs (*p* < 0.001; three-way ANOVA, circles represent individual data points’). ***p < 0.001.

We performed whole-cell patch clamp recordings in brain slices from CA1 cells of dorsal hippocampus in male WT and KO littermate animals (6–8 weeks old). Somatic current injection was used to inject various waveforms of current (Fig. 1*A*). These included a square current step, frozen noise riding on a current step, and a sinusoidal wave form riding on a current step (Fig. 2). In each case, we measured the variability in spiking over the 900 ms of current injection, in the following two respects: first, the variability within a neuron, over repeated trials (CV*_w_*); and, second, the variability between neurons, comparing matching trials between cells (CV*_b_*; see Materials and Methods).

We computed the co-efficient of variation (CV*_w_*) of spike numbers for each of the current stimulus protocols (see Materials and Methods). We observed that the CV of spike numbers across trials within cells (CV*_w_*) was significantly higher for KO cells compared with WT cells, but only for the step current protocol (CV*_w_* for WT, 0.038 ± 0.005, *n* = 30 cells; CV*_w_* for KO, 0.057 ± 0.006, *n* = 29 cells; *p* = 0.0048; Wilcoxon rank sum test; Fig. 1*B–D*). For other protocols, where frozen current noise or sinusoidal waveforms were riding on top of a current pulse, spikes were very reliable over different trials for both WTs and KOs (Fig. 2*A–E*). We then estimated the variability between cells (CV*_b_*). To do this, we compared spike numbers between different WT and KO cells by matching corresponding trials, such that we compared spiking between the first trials of WT between all the WT cells, second trials of WT between all cells, and so on forth, within the same genotype. Similar comparisons were made for KO cells as well (see Materials and Methods). From this procedure, we found that between-cell variability was significantly elevated in KO cells compared with WT cells (CV*_b_* for WT cells, 0.29 ± 0.003, *n* = 30 cells; CV*_b_* for KO cells, 0.41 ± 0.002, *n* = 29 cells; *p* < 0.001; Wilcoxon rank sum test; Fig. 1*F*).We plotted the CV*_w_* of spike numbers within the cell for WT and KO populations and observed that KOs have a much wider spread compared with WT (SD of the CV*_w_* population for WT cells = 0.026, *n* = 30 cells; SD of the CV*_w_* population for KO cells = 0.03, *n* = 29 cells; Fig. 1*G*,*H*). This implied that KO cells have a more heterogeneous population than WT cells. In KO mice, some cells were reliable (i.e., they produced a similar number of spikes across multiple trials but some did not). Based on this analysis (Fig. 1*G*,*H*), we found that 51.7% of KO cells had CV*_w_* values that did not overlap with those of WT cells.

Spike timing precision was computed by finding CV*_s_* in ISIs (Materials and Methods) and by jitter slope analysis (Materials and Methods). We compared CV*_s_*_0_ with CV*_s_*_500_ to assess the contribution of SK channels, which are shut at *t* = 0, but open by *t* = 500. We chose not to use the last ISI for this comparison because the timing of the last spike was very variable in KO cells. We then compared CV*_s_* at *t* = 0 and *t* = 500, between WT and KO cells using two-way ANOVA. We found that there was no significant difference between WT and KO cells for CV*_s_*_0_. However, at *t* = 500, we found that CV*_s_*_500_KO_ was significantly elevated compared with CV*_s_*_500_WT_ (CV_s0_WT_ = 0.11 ± 0.014; CV_s0_KO_ = 0.09 ± 0.007; CV*_s_*_500_WT_ = 0.13 ± 0.01; CV*_s_*_500_KO_ = 0.23 ± 0.03; *F*_(1,113)_ = 5.78; *p* = 0.018 for WT versus KO differences; two-way ANOVA, *t* test with Bonferroni corrections; Fig. 1*J*). The jitter slope analysis did not report a significant difference in spike imprecision between WT and KO. We used the last 400 ms of the trial for the analysis as this part showed peak SK current activity. The analysis was weakened because of our criterion that a cell should have minimum of two spikes for regression fit. Hence, several KO cells were rejected, although these were probably the ones that probably had the strongest effect of elevated SK channel activity (Fig. 1*K*,*L*).

We observed increased within-cell variability (i.e., CV*_w_*), between-cell variability (i.e., CV*_b_*) of spike numbers and increased CV*_s_*_500_ of spike timings, which are all indicative of abnormal coding in KO neurons. Thus, these initial recordings showed that there was increased heterogeneity in spiking in CA1 pyramidal neurons of KO mice.

### KO mice exhibit increased CV*_b_* for frozen noise stimuli

Neuronal coding takes place against a noisy background of continuous synaptic input (Faisal et al., 2008). Frozen noise stimuli have therefore been used in several studies to examine single-neuron computations and are considered more physiological (Stevens and Zador, 1998; Carandini, 2004; Fujisawa et al., 2004; Han and Boyden, 2007). We injected noise waveforms in the soma using a design similar to that of Mainen and Sejnowski (1995). The stimulus waveforms were mainly either of random noise or sinuosoidal input currents (for details, see Materials and Methods). The RMP was controlled by maintaining constant current injection into the cell while recording. All cells were held at a constant potential of −65 mV for all of the protocols. Cells were accepted only if the fluctuations in their potential while recordings were smaller than ±2.5 mV. For noisy inputs, spikes across multiple trials aligned very precisely for both WT cells (Fig. 2*A*,*C*) and KO cells (Fig. 2*B*,*D*). On computation of CV of spike numbers across trials within a cell (i.e., CV*_w_*), there were no statistically significant differences for any of the protocols (Fig. 2*E*). However, between-cell variability differences (CV*_b_*) were still significantly elevated for KO cells, for all stimulus protocols (Fig. 2*F*). We performed a three-way ANOVA for all of the protocols on the number of spikes produced in respective protocols for WT and KO cells. The three factors were as follows: across trials, across cells, and WT versus KO cells. ANOVA results were significant across cells (*p* < 0.001) and for WT versus KO (*p* < 0.001), but not for across trials (*p* = 1) for all the protocols. Thus, between-cell variability (CV*_b_*) reveals robust phenotypic differences, which are also observed between WT and KO cells for the input noise stimulus. The same is not true for across-trial within-cell (CV*_w_*) differences.

### Spike imprecision diverges between KO and WT only in adult mice

**Figure 3.**
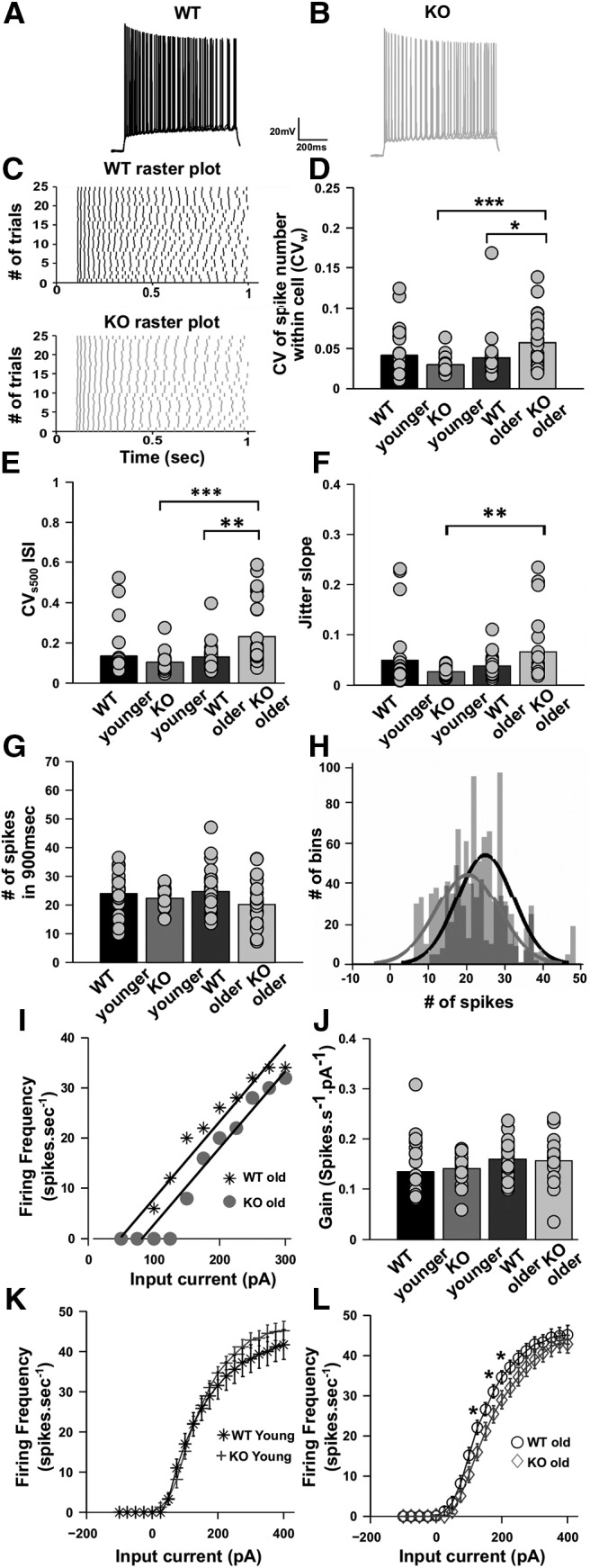
Spiking variability and spike imprecision diverges in adult KO animals compared with younger KO animals, but not in WT animals. ***A***, ***B***, Representative raw traces of spiking, superimposed for four trials only for WT younger (black) and KO younger (gray). Calibration: 20 mV, 200 ms. ***C***, Representative raster plots showing spiking for the step depolarization protocol for WT younger (black) and KO younger (gray) animals. Spikes align precisely and have low within-cell variability (CV*_w_*) across trials for KO younger compared with WT younger animals. ***D***, Bar plot for comparing within-cell variability (CV*_w_*) across multiple trials among WT younger (*n* = 26 cells, black), KO younger (*n* = 19 cells, gray), WT older (*n* = 30 cells, light black), and KO older (*n* = 29 cells, light gray) animals. Spiking variability significantly increases for KO animals with age but not for WT animals (*p* = 0.005 for interaction factor between age and genotype; two-way ANOVA). ***E***, Bar plot for CV*_s_*_500_ for the ISI of the step input protocol, a measure of spike precision for all groups. Spiking precision significantly decreases for KO animals with age but not for WT animals (*p* = 0.003 for interaction factor between age and genotype; two-way ANOVA). ***F***, Bar plot for jitter slope analysis, a measure of spike precision for all groups, as mentioned in ***E***. Spiking precision significantly decreases for KO animals with age but not for WT animals. The only significant differences emerge between KO young and KO old animals (*F*_(1,92) =_ 6.83; *p* = 0.011 for interaction factor between age and genotype; two-way ANOVA; error bars represent the SEM). ***G***, Comparison of the number of spikes produced during the 900 ms stimulus period for each of the groups in ***E***. There is a nonsignificant change in spike number with age in WT or KO animals (*p* = 0.6; two-way ANOVA), but there was a significant reduction in spike number for KO animals compared with WT animals (*p* = 0.021; two-way ANOVA). ***H***, Superimposed histograms for the number of spikes produced for WT older (light gray) and for KO older (dark gray) animals. Solid lines represent Gaussian fitting. The shift in peaks of the histograms shows that there is a greater spread in spike numbers produced in given trials for KO compared with WT animals (*p* < 0.001; three-way ANOVA). ***I***, Illustrative f–I curves for a representative cell from older (>6 weeks of age) WT animals (black stars) and a cell from older KO animals (filled gray circles). The gain (i.e., slope) for each cell was computed using a regression fit in the approximately linear domain between 50 and 300 pA. ***J***, Gain computed by fitting a regression line through the f–I curve for each cell as illustrated in ***I***, for each of the four experimental conditions. The cells were the same as in ***G***. The gains are not significantly different between WT and KO animals in any age group (*n* = 27 cells for WT older and *n* = 25 cells for KO older animals; *n* = 26 cells for WT younger and *n* = 19 cells for KO younger animals; *F*_(1,94)_ = 0.03; *p* = 0.86 for WT and KO differences; two-way ANOVA). ***K***, f–I curve for younger WT (black stars, *n* = 26 cells) versus younger KO (gray plus,19 cells) CA1 cells. There is no significant difference between WT and KO cells at this age (*F*_(1,923)_ = 0.92; *p* = 0.33 for WT and KO differences; two-way ANOVA, error bar represents the SEM). ***L***, f–I curve for older WT (black circles, *n* = 27 cells) versus older KO (gray diamonds, 25 cells) CA1 cells. There is a significant difference between WT and KO cells at this age (*F*_(1,1070)_ = 31.03; *p* < 0.001 for WT and KO differences; two-way ANOVA, error bar represents the SEM). ***p < 0.001, **p < 0.01, *p < 0.05.

Fragile X is known to have an age-dependent effect where some disease phenotypes get worse with age (Larson, 2005; Cornish et al., 2008; Choi et al., 2010). We asked whether similar effects of age might manifest in the phenotype of the firing imprecision of KO cells. We therefore repeated our experimental protocols in young (3- to 4-week-old) male WT and KO littermate mice. Regular spike firing was observed in both KO and WT cells for step input current injection (Fig. 3*A–C*). As before, we assessed the variability of spike numbers across multiple trials within the cell (CV*_w_*).There was no significant difference between WT and KO cells in these younger mice (CV*_w_* for WT, 0.041 ± 0.006, *n* = 26 cells; CV*_w_* for KO, 0.03 ± 0.002, *n* = 19 cells; *p* = 0.81, Wilcoxon rank sum test; Fig. 3*A–D*). Spike precision was assessed as above by finding the CV*_s_*_500_. By this measure too, no significant difference in the precision of spike timings was found for KO compared with WT (CV*_s_*_500_WT younger_, 0.133 ± 0.02, *n* = 26 cells; CV*_s_*_500_KO younger_, 0.103 ± 0.01, *n* = 19 cells; *p* = 0.82, Wilcoxon rank sum test; Fig. 3*E*). We used a two-way ANOVA interaction model for assessing age-dependent differences in spiking variability across different trials in a cell (CV*_w_*; *F*_(1,100)_ = 4.97, *p* = 0.028 for WT and KO differences; *F*_(1,100)_ = 8.05, *p* = 0.005 for interaction factor; two-way ANOVA with interaction model, *t* test with Bonferroni corrections *post hoc*; Fig. 3*D*). We also estimated differences in spike timing precision across different trials in a cell (CV*_s_*_500_; *F*_(1,99)_ = 7.47, *p* = 0.007 for age factor; *F*_(1,99)_ = 9.46, *p* = 0.003 for interaction factor; two-way ANOVA with interaction model, *t* test with Bonferroni corrections; Fig. 3*E*). Spike timing precision differences were also assessed by jitter slope analysis (*F*_(1,92)_ = 6.83, *p* = 0.011 for interaction factor; *F*_(1,92)_ = 2.2, *p* = 0.14 for age factor; two-way ANOVA with interaction model, *t* test with Bonferroni corrections; Fig. 3*F*). ANOVA indicated a significant interaction between the two factors age and genotype of the animal. Using *post hoc* tests, we concluded that for KO cells there is a worsening of phenotype for both variability in spiking number (CV*_w_*) across multiple trials within a cell and in spike timing precision (CV*_s_*_500_ of ISI) across multiple trials within a cell (Fig. 3*D–F*; Table 1, detailed statistics). However, the same was not seen in WT cells. A two-way ANOVA was performed on the number of spikes produced in the 900 ms stimulus period used in the step depolarization protocol (Fig. 1*A*). In this ANOVA, the two factors were young versus old animals and WT versus KO. There was no significant change between the ages of the animals for both WT and KO (*F*_(1,10)_ = 0.31; *p* = 0.6; two-way ANOVA, *t* test with Bonferroni correction), but there was a significant reduction in spike number for KO animals compared with WT animals (*F*_(1,101)_ = 4.7; *p* = 0.032; two-way ANOVA, *t* test with Bonferroni correction; Fig. 3*G*). However, the f–I curves were not significantly different between WT and KO animals in either in the older (8 weeks) or younger (4 weeks) age groups, when the slope of the f–I curves (gain) was used as a readout (mean gain for f–I curve for older WT animals, 0.16 ± 0.007 spikes · s^−1^ · pA^−1^, *n* = 27 cells; mean gain for f–I curve for older KO animals, 0.16 ± 0.008 spikes · s^−1^ · pA^−1^, *n* = 25 cells; mean gain for f–I curve for younger WT animals, 0.133 ± 0.01 spikes · s^−1^ · pA^−1^, *n* = 26 cells; mean gain for f–I curve younger KO animals, 0.136 ± 0.013 spikes · s^−1^ · pA^−1^, *n* = 19 cells; *F*_(1,94)_ = 0.03, *p* = 0.86 for WT and KO differences; two-way ANOVA, *t* test with Bonferroni correction; Fig. 3*I*,*J*). When comparisons were made of f–I curves of WT and KOs cells, there was reduced excitability seen for some input currents in older animals (mean number of spikes for an input current of 125 pA in WT animals, 22.07 ± 1.74; *n* = 27 cells; mean number of spikes for input current of 125 pA in KO animals, 15.92 ± 2.1; *n* = 25 cells; *F*_(1,1070)_ = 31.03, *p* < 0.001; two-way ANOVA; Fig. 3*L*), but not in younger animals (mean number of spikes for input current of 125 pA in WT animals, 21.8 ± 2.9; *n* = 26 cells; mean number of spikes for input current of 125 pA in KO animals, 22.8 ± 2.9; *n* = 19 cells, *F*_(1,923)_ = 0.92, *p* = 0.33; two-way ANOVA, *t* test with Bonferroni correction as *post hoc*; Fig. 3*K*). Other properties like spike threshold were not significantly different between WT and KO cells (spike threshold for the first spike in WT, −53.6 ± 0.56 mV, *n* = 30 cells; spike threshold for first spike KO, −53.8 ± 0.62 mV, *n* = 29 cells; spike threshold for the last spike WT, −41.9 ± 0.66 mV, *n* = 30 cells; spike threshold for the last spike KO, −43.5 ± 0.66 mV, *n* = 29 cells; *F*_(1,115)_ = 1.98, *p* = 0.16, two-way ANOVA, *t* test with Bonferroni correction as *post hoc* test. The spread in the number of spikes produced by KO neurons was greater than observed in WT neurons, further substantiating the point of increased variability in KO neurons (SD of spike numbers for WT = 7.2; *n* = 750 spikes from 30 cells 25 trials each; SD of spike numbers for KO = 8.1; *n* = 725 spikes from 29 cells 25 trials each; Fig. 3*H*). We performed a three-way ANOVA in which the factors were as follows: across trials, across cells, and WT versus KO for older age group data. The ANOVA results were highly significant for the variability in spike numbers between cells (CV*_b_*; *F*_(29,1420)_ = 32.04, *p* < 0.001) and differences in spike numbers between WT and KO for older animals (*F*_(1,1420)_ = 209.9, *p* < 0.001), but across-trial comparisons did not exhibit a significant difference (*F*_(24,1420)_ = 0.15, *p* = 1).Thus, there is an increase in spike variability with lowered spiking precision for adult KO mice compared with juvenile KO mice. This divergence that is observed for KO animals with age does not happen for WT animals.

**Table 1:** Detailed statistics used in different figures

Figure number	Data structure	Type of test	Statistics
Figure 1*E*	Non-normal	Wilcoxon rank sum test	*p* = 0.0048 for CV*_w_* differences between WT older and KO older
Figure 1*F*	Non-normal	Wilcoxon rank sum test	*p* < 0.001 for CV*_b_* differences between WT older and KO older
Figure 1*J*	Non-normal	Two-way ANOVA	*F*_(1,113)_ = 5.78; *p* = 0.018 for WT vs KO differences
Figure 1*L*	Non-normal	Wilcoxon rank sum test	*p* = 0.14 for jitter slope differences between WT older and KO older
Figure 2*E*	Normal	Three-way ANOVA	*p* < 0.001 for CV*_w_* differences between WT older and KO older
Figure 2*F*	Normal	Three-way ANOVA	*p* < 0.001 for CV*_b_* differences between WT older and KO older
Figure 3*D*	Normal	Two-way ANOVA	*F*_(1,100)_ = 4.97; *p* = 0.028 for WT and KO differences; *F*_(1,100)_ = 8.05; *p* = 0.005 for interaction factor between age and genotype; *p* = 0.7, for WT older vs WT younger; *p* < 0.001 for KO older vs KO younger, *t* test with Bonferroni correction
Figure 3*E*	Normal	Two-way ANOVA	*F*_(1,99)_ = 7.47, *p* = 0.007 for age factor; *F*_(1,99)_ = 9.46; *p* = 0.003 for interaction factor between age and genotype; *p* = 0.0002 for KO older vs KO younger; *p* = 0.9 for WT older vs WT younger; *p* = 0.0025 for WT older and KO older
Figure 3*F*	Normal	Two-way ANOVA	*F*_(1,92)_ = 6.83, *p* = 0.011 for interaction factor between age and genotype, *F*_(1,92)_ = 2.2, *p* = 0.14 for age factor; *p* = 0.052 for WT older and KO older; *p* = 0.007 for KO older and KO younger; *p* = 0.4for WT older and WT younger
Figure 3*G*	Normal	Two-way ANOVA	*F*_(1,100)_ = 4.71; *p* = 0.032 for spike number between WT and KO; *p* = 0.029 between WT old and KO old; *p* = 0.3 between WT young and KO young
Figure 3*J*	Normal	Two-way ANOVA	*F*_(1,94)_ = 0.03; *p* = 0.86 for WT and KO differences
Figure 3*K*	Normal	Two-way ANOVA	*F*_(1,923)_ = 0.92; *p* = 0.33; WT and KO differences at young age group
Figure 3*L*	Normal	Two-way ANOVA	*F*_(1,1070)_ = 31.03 *p* < 0.001; WT and KO differences at old age group
Figure 4*B*	Normal	Two-way ANOVA	*F*_(1,72)_ = 5.35; *p* = 0.023 for difference between WT and KO CA3 cells younger animals
Figure 4*D*	Normal	Two-way ANOVA	*F*_(1,88)_ = 1.06; *p* = 0.31 for difference between WT and KO CA1 cells younger animals
Figure 4*F*	Normal	Two-way ANOVA	*F*_(1,75)_ = 1.16; *p* = 0.28 for difference between WT and KO CA3 cells older animals
Figure 4*H*	Normal	Two-way ANOVA	*F*_(1,59)_ = 7.42; *p* = 0.009 for difference between WT and KO CA1 cells for older animals
Figure 5*C*	Normal	Two-way ANOVA	*F*_(1,46)_ = 4.8; *p* = 0.033 for mAHP WT and KO differences. *p* = 0.2 between WT younger and KO younger; *p* = 0.018 for WT older and KO older
Figure 5*D*	Normal	Two-way ANOVA	*F*_(1,54)_ = 8.84; *p* = 0.004 for sAHP WT and KO differences; *p* = 0.07 between WT younger and KO younger; *p* = 0.008 for WT older and KO older
Figure 5*F*	Non-normal	Wilcoxon rank sum test	*p* = 0.91 for *I*_h_ percentage of sag between WT and KO
Figure 5*H*	Normal	Two-way ANOVA	*F*_(1,258)_ = 2.54; *p* = 0.11 between WT and KO for I_M_ current
Figure 6*C*	Normal	Two-way ANOVA	*F*_(1,240)_ = 33.3; *p* < 0.001 for WT older and KO older SK currents
Figure 6*D*	Normal	Two-way ANOVA	*F*_(1,189)_ = 0.51; *p* = 0.5 for WT younger and KO younger SK currents
Figure 6*E*	Normal	Two-way ANOVA	*F*_(1,229)_ = 0.01; *p* = 0.9 for WT older and WT younger SK currents
Figure 6*F*	Normal	Two-way ANOVA	*F*_(1,209)_ = 21.02; *p* < 0.001 for KO older and KO younger SK currents
Figure 7*B*	Normal	Two-way ANOVA	*F*_(1,24)_ = 0.48; *p* = 0.5 for WT younger vs KO younger for CA3
Figure 7*C*	Normal	Two-way ANOVA	*F*_(1,24)_ = 0.21; *p* = 0.6 for WT younger vs KO younger for CA1
Figure 7*D*	Normal	Two-way ANOVA	*F*_(1,24)_ = 0.3; *p* = 0.6 for WT older vs KO older for CA3
Figure 7*E*	Normal	Two-way ANOVA	*F*_(1,24)_ = 0.25; *p* = 0.61 for WT older vs KO older for CA1
Figure 8*E*	Normal	Two-way ANOVA	*F*_(1,48)_ = 0; *p* = 0.96 for effect of apamin; *F*_(1,49)_ = 8.74; *p* = 0.005 for WT and KO differences
Figure 8*F*	Normal	Two-way ANOVA	*F*_(1,96)_ = 157.5; *p* < 0.001 for WT and KO differences; *F*_(1,96)_ = 53.1; *p* < 0.001 for effect of apamin on CV*_b_*
Figure 8*G*	Normal	Two-way ANOVA	*F*_(1,49)_ = 9.63; p=0.003 for WT and KO differences; *F*_(1,49)_ = 2.87; *p* = 0.09 for effect of apamin; *p* = 0.006 for WT pre apamin and KO pre apamin; *p* = 0.05 for KO pre apamin and KO post apamin; *p* = 0.23 WT pre apamin and KO post apamin
Figure 8*H*	Normal	Two-way ANOVA	*F*_(1,41)_ = 2.1; *p* = 0.15 for effect of apamin; *F*_(1,41)_ = 3.6; *p* = 0.06 for WT and KO differences
Figure 8*I*	Normal	Two-way ANOVA	*F*_(1,81)_ = 2.7; *p* = 0.1 for differences between WT and KO; *F*_(1,81)_ = 40.5 *p* < 0.001 for effect of BAPTA

**Figure 4.**
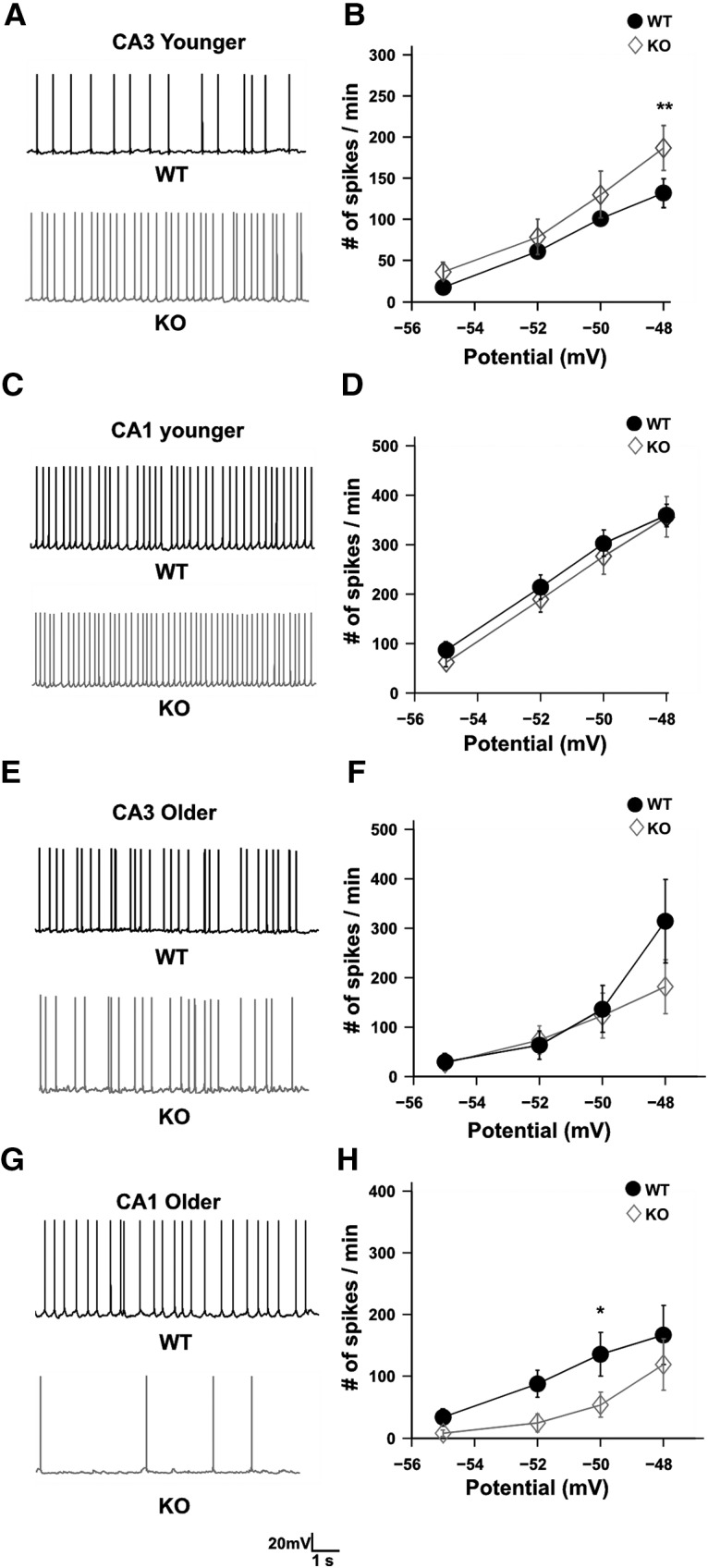
Contrasting effects of FMRP KO in different regions of hippocampus. ***A***, Representative traces showing increased excitability between WT (black) and KO (gray) CA3 cells in younger animals. Traces are from the recording where the cells are held at −48 mV. Calibration: 20 mV, 1 s. ***B***, Line plot showing increased excitability for KO CA3 cells (*n* = 10 cells, gray diamond) compared with WT CA3 cells (*n* = 10 cells, filled black circles), when the cells were held at −48 mV, for younger animals. For other holding potentials, the spike numbers are not significantly increased for KO cells (*F*_(1,72)_ = 5.35, *p* = 0.023 for difference between WT and KO; two-way ANOVA, error bar represents the SEM). ***C***, Representative traces showing no change in excitability between WT (black) and KO (gray) CA1 cells in younger animals. Traces are from the recording where the cells are held at −48 mV. Calibration: 20 mV, 1 s. ***D***, Line plot showing no change in excitability for KO CA1 cells (*n* = 12 cells, gray diamond) compared with WT CA1 cells in younger animals (*n* = 12 cells, filled black circles; *F*_(1,88)_ = 1.06, *p* = 0.31 for difference between WT and KO; two-way ANOVA, error bar represents the SEM). ***E***, Representative traces showing no change in excitability between WT (black) and KO (gray) CA3 cells in older animals. Traces are from the recording where the cells are held at −48 mV. Calibration: 20 mV, 1 s. ***F***, Line plot showing no change in excitability for KO CA3 cells (*n* = 10 cells, gray diamond) compared with WT CA3 cells in older animals (*n* = 10 cells, filled black circles; *F*_(1,72)_ = 1.17, *p* = 0.28 for difference between WT and KO; two-way ANOVA, error bar represents the SEM). ***G***, Representative traces showing reduced excitability for KO cells (gray) compared with (black) and KO CA1 cells in older animals. Traces are from the recording where the cells are held at −50 mV. Calibration: 20 mV, 1 s. ***H***, Line plot showing decreased excitability for KO CA1 cells (*n* = 8 cells, gray diamond) compared with WT CA1 cells (*n* = 8 cells, filled black circles), when the cells were held at −50 mV, for older animals. For other holding potentials, the spike numbers are not significantly decreased for KO cells (*F*_(1,59)_ = 7.42, *p* = 0.009 for difference between WT and KO; two-way ANOVA, error bar represents the SEM). **p < 0.01, *p < 0.05.

It is to be noted that all of the data in figures is from CA1 cells except in Figure 4, where it is from both CA3 and CA1 cells

### Differential effect of FMRP KO in different brain regions

Mouse models of FXS are known to exhibit increased excitability (Contractor et al., 2015; Deng and Klyachko, 2016; Luque et al., 2017). In contrast to previous studies, we observed that excitability was reduced in CA1 neurons in adult mice, but not in juveniles. Deng et al. (2019) have observed increased CA3 excitability in juveniles, which was in contrast to what we observed in CA1, namely, that there is no significant effect on excitability of CA1 pyramidal cells at this time point. To address this contrasting result, we repeated the protocol from Deng et al. (2019) in our FXS model and compared outcomes between CA3 and CA1 (Fig. 4). In agreement with their study, we observed significantly increased spiking in CA3 cells of FXS mice for some of the input holding currents (number of spikes in 60 s for −48 mV holding potential for WT, 131.9 ± 17.42, *n* = 10 cells; number of spikes in 60 s for −48 mV holding potential for KO, 186.3 ± 27.27, *n* = 10 cells; *F*_(1,72)_ = 5.35, *p* = 0.023 for difference between WT and KO; two-way ANOVA, Sidak’s *post hoc* test; Fig. 4*A*,*B*). We performed similar experiments in CA1 pyramidal cells, using the same protocol as in Figure 4, *A* and *B*. We did not find any difference in excitability between WT and KO (number of spikes in 60 s for −48 mV holding potential for WT, 355.58 ± 40.93, *n* = 12 cells; number of spikes in 60 s for −48 mV holding potential for KO, 358.67 ± 22.56, *n* = 12 cells; *F*_(1,88)_ = 1.06, *p* = 0.31 for difference between WT and KO; two-way ANOVA, Sidak’s *post hoc* test; Fig. 4*C*,*D*). On seeing this differential effect of FMRP KO in different regions of hippocampus, we were interested to see whether this might be a function of age. Therefore, we recorded similar protocols from CA3 and CA1 of older animals. We found that the hyperexcitability seen in younger CA3 cells goes away, leading to no significant difference in the spiking activity in these cells for WT versus KO (number of spikes in 60 s for −48 mV holding potential for WT, 313.6 ± 83.8, *n* = 10 cells; number of spikes in 60 s for −48 mV holding potential for KO, 181.1 ± 54.9, *n* = 10 cells; *F*_(1,75)_ = 1.16, *p* = 0.28 for difference between WT and KO; two-way ANOVA, Sidak’s *post hoc* test; Fig. 4*E*,*F*). In agreement with our previous results, as shown in Figure 3, we observed reduced excitability in CA1 KO cells compared with WT cells (number of spikes in 60 s for −50 mV holding potential for WT, 135.6 ± 35.2, *n* = 8 cells; number of spikes in 60 s for −50 mV holding potential for KO, 54 ± 20.4, *n* = 8 cells; *F*_(1,59)_ = 7.42, *p* = 0.009 for difference between WT and KO; two-way ANOVA, Sidak’s *post hoc* test; Fig. 4*G*,*H*). Using these experiments, we were able to replicate previous published results of Deng et al. (2019). In addition, we found that there were contrasting and age-dependent effects of FMRP KO on the spiking properties of CA1 and CA3 pyramidal neurons.

### mAHP currents are elevated in older KO animals compared with WT but no significant changes in *I*_h_ and M currents

We next investigated the mechanisms for increased variability in KO animals, on the basis of the above observations of increased variability and fewer spikes (Fig. 3*D*,*G*,*L*) in the later part of the current pulse. In particular, we hypothesized that mAHP and sAHP currents were likely candidates to mediate such slower-onset effects. We asked whether these currents might be altered in FXS cells.

**Figure 5.**
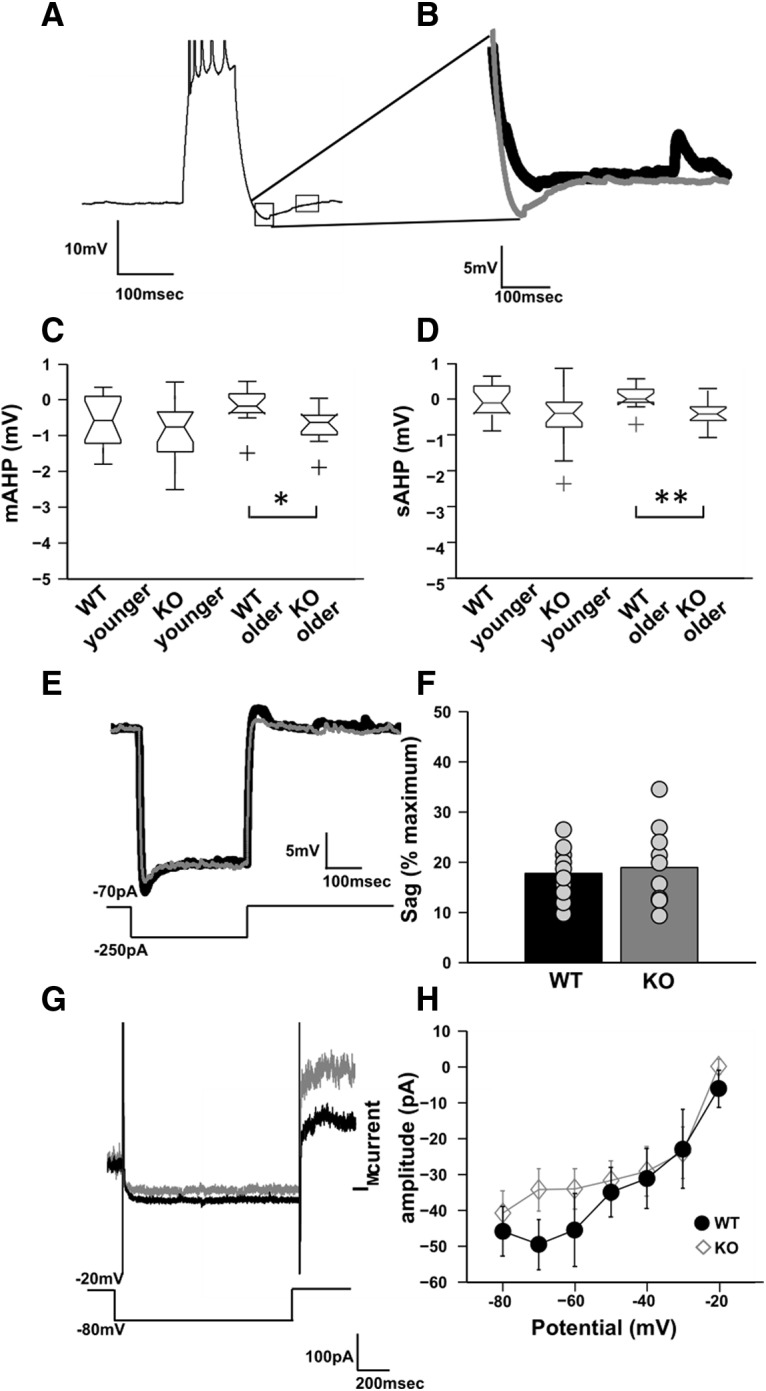
mAHP and sAHP are elevated in FXS KO neurons. ***A***, Representative trace for the protocol to measure mAHP and sAHP. Hyperpolarization within 50 ms of the spike train (1) is quantified as mAHP, while hyperpolarization following the spike train 200 ms after the spike train (2) is quantified as sAHP. Calibration: 10 mV, 100 ms. ***B***, Superimposed traces of WT older and KO older AHP currents showing elevated mAHP currents for KO cell (gray) compared with WT cell (black). Calibration: 5 mV, 100 ms. ***C***, Box plot showing mAHP currents for all groups (*n* = 12 cells for WT older animals, *n* = 13 cells for KO older animals, *n* = 14 cells for WT younger animals, and *n* = 18 cells for KO younger animals). mAHP values are elevated only for older KO animals versus older WT animals (*p* = 0.033, two-way ANOVA, plus sign denotes an outlier). ***D***, Box plot showing sAHP currents for all groups (*n* = 12 cells for WT older animals, *n* = 13 cells for KO older animals, *n* = 14 cells for WT younger animals, and *n* = 18 cells for KO younger animals). sAHP values are elevated only for older KO animals versus older WT animals (*p* = 0.004, two-way ANOVA, plus sign denotes an outlier). ***E***, Representative trace for the protocol to measure *I*_h_ voltage deflection. Superimposed traces of WT (black) and KO (gray) showing no significant difference in the response. Sag produced in response to 250 pA hyperpolarizing pulse was quantified to estimate *I*_h_ voltage deflection. Calibration: 5 mV, 100 ms. ***F***, Bar plot for showing the percentage of sag produced due to *I*_h_ voltage deflection for WT older and KO older animals (*n* = 10 cells for both WT and KO older animals). There was no significant difference in sag due to *I*_h_ current (*p* = 0.91, Wilcoxon rank sum test). ***G***, Representative traces showing XE991-sensitive M current for WT older (black) and KO older (gray) animals. The traces were plotted by finding the difference between pre- and postblocker current trace for an input step from −20 to −80 mV. Calibration: 100 pA, 200 ms. ***H***, Line plot showing the *I–V* curve of XE 991-sensitive M current. There is no significant difference in the M currents between WT older (filled black circles) and KO older (gray diamond) animals for any of the input steps (*n* = 19 cells for both WT and KO, *F*_(1,258)_ = 2.54, *p* = 0.11 between WT and KO; two-way ANOVA, error bars represent the SEM). **p < 0.01, *p < 0.05.

To test this hypothesis, we compared AHPs for WT versus KO in older and younger animals. Cells were held at −70 mV to record AHPs. We quantified AHPs by examining the hyperpolarization elicited with a spike train of five spikes in 100 ms. Hyperpolarization was quantified at mAHP peak (50 ms after the spike train) and at sAHP peak (200 ms after the spike train). We found that mAHP was elevated only in older KOs but not in younger KOs when compared with their WT littermates (mAHP for older WTs, −0.18 ± 0.14, *n* = 12 cells; mAHP for older KOs, −0.71 ± 0.14; *n* = 13 cells; mAHP for younger WTs, −0.6 ± 0.19, *n* = 14 cells; mAHP for younger KOs, −1.04 ± 0.4, *n* = 18 cells; *F*_(1,46)_ = 4.8, *p* = 0.033 for WT and KO differences; two-way ANOVA, *t* test with Bonferroni correction; Fig. 5*C*, Table 1, detailed statistics) . We found similar results for sAHP measurements as well. We found that sAHP was significantly elevated only for older KO animals compared with their WT littermates but not significantly different for younger KO animals compared with their younger WT littermates (sAHP for older WTs, 0.05 ± 0.09, *n* = 12 cells; sAHP for older KOs, 0.4 ± 0.11; *n* = 13 cells; sAHP for younger WTs, 0.07 ± 0.12, *n* = 14 cells; sAHP for younger KOs, 0.5 ± 0.17, *n* = 18 cells; *F*_(1,54)_ = 8.84, *p* = 0.004 for WT and KO differences; two-way ANOVA, *t* test with Bonferroni correction; Fig. 5*D*). As there was no significant difference in spike variability and spike time precision in younger KO animals, we hypothesized that the observed spiking variability in older KO animals might be due to elevated mAHPs, which has a strong change with age in KO animals. Further, the stimulus period used in the previous experiments (Fig. 1) was of 900 ms, which is the peak time of activation for mAHP mediating ion channels.

The mAHP in CA1 cells is mediated by *I*_h_**_,_**M, and SK currents. A previous study has shown that *I*_h_ voltage sag is not significantly altered in soma of FXS cells (Brager et al., 2012). M currents are known to affect the RMP and spike adaptation index (Madison et al., 1987; Guan et al., 2011; Nigro et al., 2014; Hönigsperger et al., 2015). Neither RMP nor spike adaptation index were significantly changed in FXS cells, indicating that M currents might not be responsible for the observed phenotype. To further test this idea, *I*_h_ voltage sag and M currents were recorded from soma of CA1 cells and compared between WT and KO cells for adult animals. As there was no difference in mAHP at juvenile stage for KO cells, we did not measure their *I*_h_ voltage sag and M currents. *I*_h_ channel activity was recorded using a hyperpolarization pulse. The voltage sag produced in response to the pulse, was quantified according to a method adapted from Brager et al. (2012; see Materials and Methods; Fig. 5*E*). As observed previously, there was no significant difference in *I*_h_ sag between WT and KO CA1 cells, at somatic level (percentage of sag for WT, 17.7 ± 1.64, *n* = 10 cells; percentage of sag for KO, 18.87 ± 2.51, *n* = 10 cells; *p* = 0.91, Wilcoxon rank sum test; Fig. 5*F*).

To measure M currents, we used the protocol adapted from Shah et al., 2002 (Materials and Methods). According to the protocol, the cells were held at −20 mV and M currents were deactivated in steps from −20 to −80 mV. M current-specific blocker XE991 was used to obtain the postblocker trace. Postblocker trace was subtracted from preblocker trace to obtain XE991-sensitive M currents (Fig. 5*G*). We found no significant differences in M currents between WT and KO CA1 cells. (mean M current for step −80 mV for WT, −40.9 ± 6.33, *n* = 19 cells; mean M current for step −80 mV for KO, 45.94 ± 6.87, *n* = 19 cells; *F*_(1,258)_ = 2.54, *p* = 0.11 between WT and KO; two-way ANOVA, *t* test with Bonferroni correction; Fig. 5*H*). Overall, these observations suggest that neither *I*_h_ voltage sag nor M currents contribute to the observed elevation in mAHP in KO animals.

### SK currents are elevated in older KO animals compared with WT

**Figure 6.**
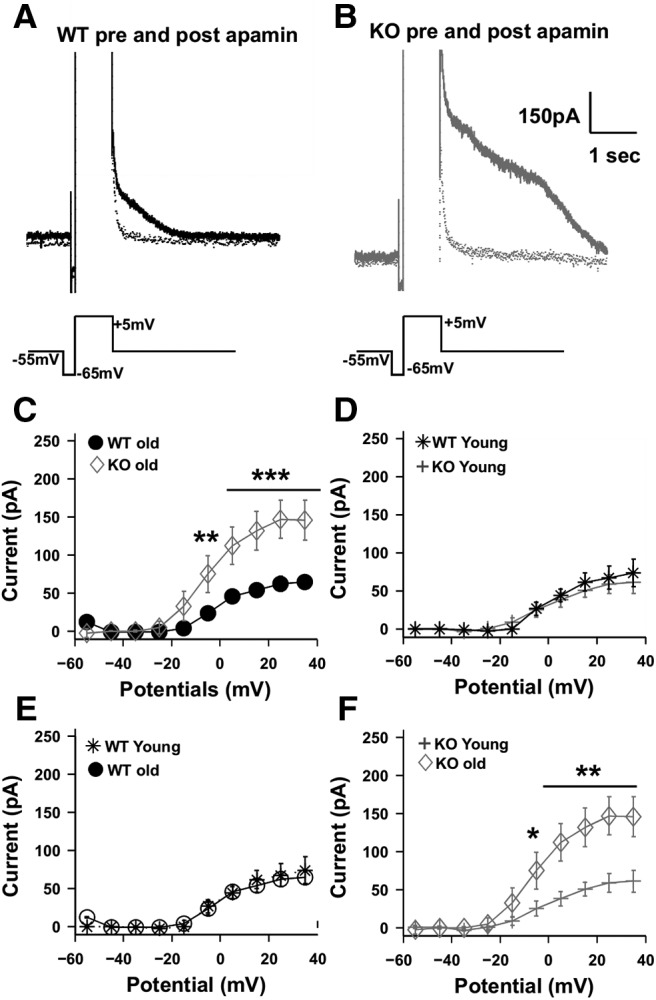
Apamin-sensitive SK currents are elevated in KO neurons compared with WT neurons. ***A***, ***B***, Representative traces showing the outward currents elicited by step depolarization voltage commands in voltage-clamp mode, for WT neuron (black; ***A***) and for KO neuron (gray), pre-apamin (solid line) and post-apamin (dotted line) perfusion (***B***). Trace below indicates command voltage protocol. Calibration: 150 pA, 1 s. ***C***, *I–V* curve of the apamin-sensitive current elicited across multiple steps for WT older cells (*n* = 13 cells, filled black circles) and KO older cells (*n* = 13 cells, gray diamond). Currents are significantly elevated for KO cells compared with WT cells, for multiple steps (*F*_(1,240)_ = 33.3, *p* < 0.001 between WT and KO, two-way ANOVA, error bars represent the SEM). ***D***, *I–V* curve of the apamin-sensitive current elicited across multiple steps in WT younger cells (*n* = 11 cells, black star) and KO younger cells (*n* = 9 cells, gray plus). Currents between KO and WT cells at this age group were not significantly different (*F*_(1,189)_ = 0.51; *p* = 0.5; two-way ANOVA, error bars represent the SEM). ***E***, *I–V* curve of the apamin-sensitive current elicited across multiple steps for WT younger cells (*n* = 11 cells, black stars) and WT older cells (*n* = 13 cells, black circles). There is no age-dependent change in SK currents for WT animals (*F*_(1,229)_ = 0.01, *p* = 0.9; two-way ANOVA, error bars represent the SEM). ***F***, *I–V* curve of the apamin-sensitive current elicited across multiple steps for KO younger cells (*n* = 9 cells, gray plus) and KO older cells (*n* = 13 cells, gray diamonds). There is an age-dependent increase in SK currents in KO animals (*F*_(1,209)_ = 21.02, *p* < 0.001; two-way ANOVA, bars represent the SEM). ***p < 0.001, **p < 0.01, *p < 0.05.

SK currents are also known to contribute substantially to mAHP (Sah and Clements, 1999; Stocker et al., 1999). It has also been shown that Ca^2+^ influx from VGCCs is elevated in FXS cells (Deng et al., 2011, 2013), indicating elevated Ca^2+^ levels intracellularly and hence increased SK channel activation. Thus, we hypothesized that SK currents are elevated in FXS cells, leading to the observed elevated mAHP in these cells. To test this, we used a procedure from the study by Carlen (1997). Outward currents were evoked using step depolarization of the cell from −55 to +5 mV and above (Fig. 6*A*,*B*), in voltage-clamp mode. Apamin was bath applied to obtain post-apamin perfusion trace. The difference in current before and after apamin perfusion was quantified as SK currents. These currents were significantly higher in KO animals compared with WT animals for the adult age group (SK current for older WTs at +15 mV step, 54.2 ± 9, *n* = 13 cells; SK current for older KOs at +15 mV step, 131.73 ± 25.5, *n* = 13 cells; *F*_(1,240)_ = 33.3, *p* < 0.001 between WTs and KOs, two-way ANOVA, *t* test with Bonferroni correction; Fig. 6*C*). However, this phenotype was seen only in adult WT versus adult KO animals. When similar experiments were performed in juveniles of both genotypes, there was no significant difference in SK currents (SK current for younger WTs at +15 mV step, 61.93 ± 11.9, *n* = 11 cells; SK current for younger KOs at +15 mV step, 51.2 ± 8.9, *n* = 9 cells; *F*_(1,189)_ = 0.51, *p* = 0.5 between WTs and KOs; two-way ANOVA, *t* test with Bonferroni correction; Fig. 6*D*). To delineate the developmental change in SK channels levels between WT and KO animals, comparisons were made within the same genotype between different age groups. There was no significant change in the levels of SK currents with age for WT animals (SK current for older WTs at +15 mV step, 54.2 ± 9, *n* = 13 cells; SK current for younger WTs at +15 mV step, 61.93 ± 11.9, *n* = 11 cells; *F*_(1,229)_ = 0.01, *p* = 0.9 between WTs and KOs; two-way ANOVA, *t* test with Bonferroni correction; Fig. 6*E*). On the contrary there was a significant increment in levels of SK currents for KO animals as they progressed from juvenile to adult stage (SK current for older KOs at +15 mV step, 131.73 ± 25.5, *n* = 13 cells; SK current for younger KOs at +15 mV step, 51.2 ± 8.9, *n* = 9 cells; *F*_(1,209)_ = 21.02, *p* < 0.001 between WTs and KOs; two-way ANOVA, *t* test with Bonferroni correction; Fig. 6*F*). Together, these observations show that there is an increase in the levels of SK currents with age in KO animals, and we note that this correlates with the worsening of spike reliability between adult and juvenile KO animals, as described in Figure 3.

### SK channel expression is unchanged in CA1 and CA3 regions for KO compared with WT animals

**Figure 7.**
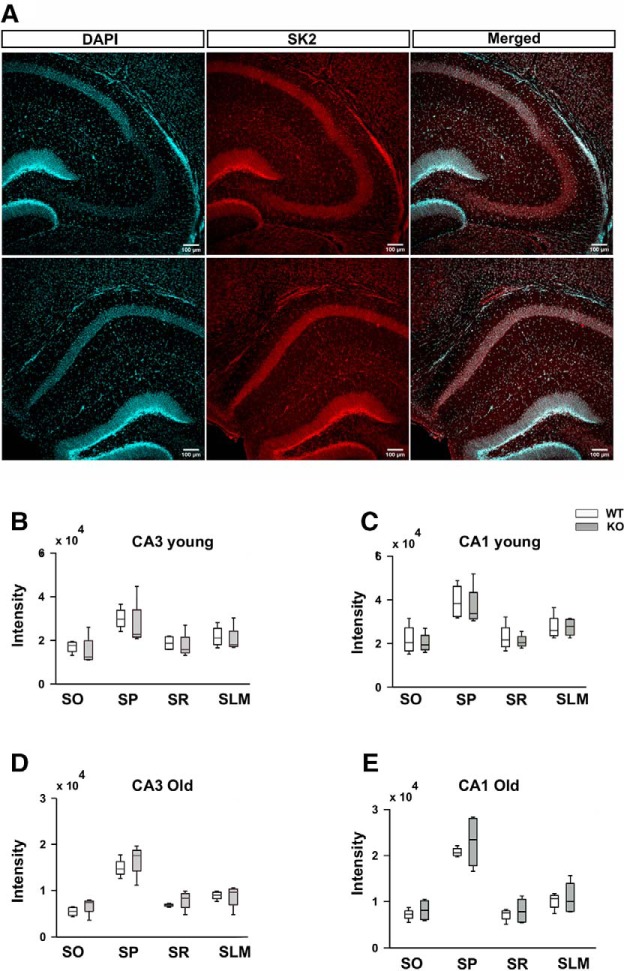
There is no significant change in SK2 channel levels in KO compared with WT animals. ***A***, Representative images of SK2 labeling in CA3 (top) and CA1 (bottom) for a WT animal. Green false color was used for DAPI for better visualization. Scale bar, 100 μm. ***B***, Analyzed box plot for mean intensity of SK2 channel in CA3 for young animals (*N* = 4 animals for WT; *N* = 4 animals for KO; *F*_(1,24)_ = 0.48; *p* = 0.5 between WT and KO; two-way ANOVA, error bars represent SEM). ***C***, Analyzed box plot for mean intensity of SK2 channel in CA1 for young animals. (*N* = 4 animals for WT; *N* = 4 animals for KO; *F*_(1,24)_ = 0.21; *p* = 0.6 between WT and KO for CA1; two-way ANOVA, error bars represent the SEM). ***D***, Analyzed box plot for the mean intensity of SK2 channel in CA3 for old animals (*N* = 4 animals for WT; *N* = 4 animals for KO; *F*_(1,24)_ = 0.3; *p* = 0.6 between WT and KO for CA1; two-way ANOVA, error bars represent the SEM). ***E***, Analyzed box plot for mean intensity of the SK2 channel in CA1 for old animals (*N* = 4 animals for WT; *N* = 4 animals for KO; *F*_(1,24)_ = 0.25; *p* = 0.61 between WT and KO for CA1; two-way ANOVA, error bars represent the SEM).

We have observed that SK current levels are elevated in KO CA1 cells compared with WT cells (Fig. 6). This elevation might arise due to an increase in the levels of SK channels or due to a change in SK channel kinetics. The latter might arise due to a change in the kinetics of the SK channel itself or due to changes in the activity of other channels that have an effect on SK channels. The putative candidates might be Ca^2+^ channels known to be affected in FXS (Meredith et al., 2007; Ferron et al., 2014; Brown et al., 2001; Contractor et al., 2015). Previous published results have not shown SK channels to be targets of FMRP for transcription regulation, decreasing the likelihood that the levels will be affected in the CA1 region (Darnell et al., 2011; Deng et al., 2019). To specifically test this point, immunofluorescence experiments were used to assess the expression of SK channels. We performed *in situ* immunolabeling to measure the intensity of the SK2 isoform in CA3 and CA1 regions of the hippocampus using a specific antibody (Materials and Methods; Fig. 7*A*). We analyzed levels of SK2 protein in different regions and subsections of the hippocampus for both WT and KO animals. Similar experiments were performed on both old (adult) and young (juvenile) slices. In young animals, we have seen an increased number of spikes in CA3 with no significant change in CA1 (Fig. 4). However, the levels of SK2 protein were not significantly altered in KO slices for either CA3 (*N* = 4 animals for WTs; *N* = 4 animals for KOs; *F*_(1,24)_ = 0.48, *p* = 0.5 for WT vs KO for CA3; two-way ANOVA, Sidak’s *post hoc* test; Fig. 7*B*) or CA1 (*N* = 4 animals for WTs; *N* = 4 animals for KOs; *F*_(1,24)_ = 0.21, *p* = 0.6 for WT vs KO cells for CA1; two-way ANOVA, Sidak’s *post hoc* test; Fig. 7*C*). For old animals, we have seen a reduction in spike number in the CA1 region (Fig. 3*G*,*L*), but no changes in SK2 levels could be found in either CA1 or CA3 (CA1: *N* = 4 animals for WTs; *N* = 4 animals for KOs; *F*_(1,24)_ = 0.25, *p* = 0.61 for WT vs KO for CA1; two-way ANOVA, Sidak’s *post hoc* test; Fig. 7*E*; CA3: *N* = 4 animals for WTs; *N* = 4 animals for KOs; *F*_(1,24)_ = 0.3; *p* = 0.6 for WT vs KO for CA3; two-way ANOVA, Sidak’s *post hoc* test; Fig. 7*D*). Overall, these labeling studies suggest that the KO does not affect SK expression in either CA1 or CA3. The latter observation is in agreement with previous results (Deng et al., 2019).

### Apamin partially rescues the phenotype of increased variability in KO animals

**Figure 8.**
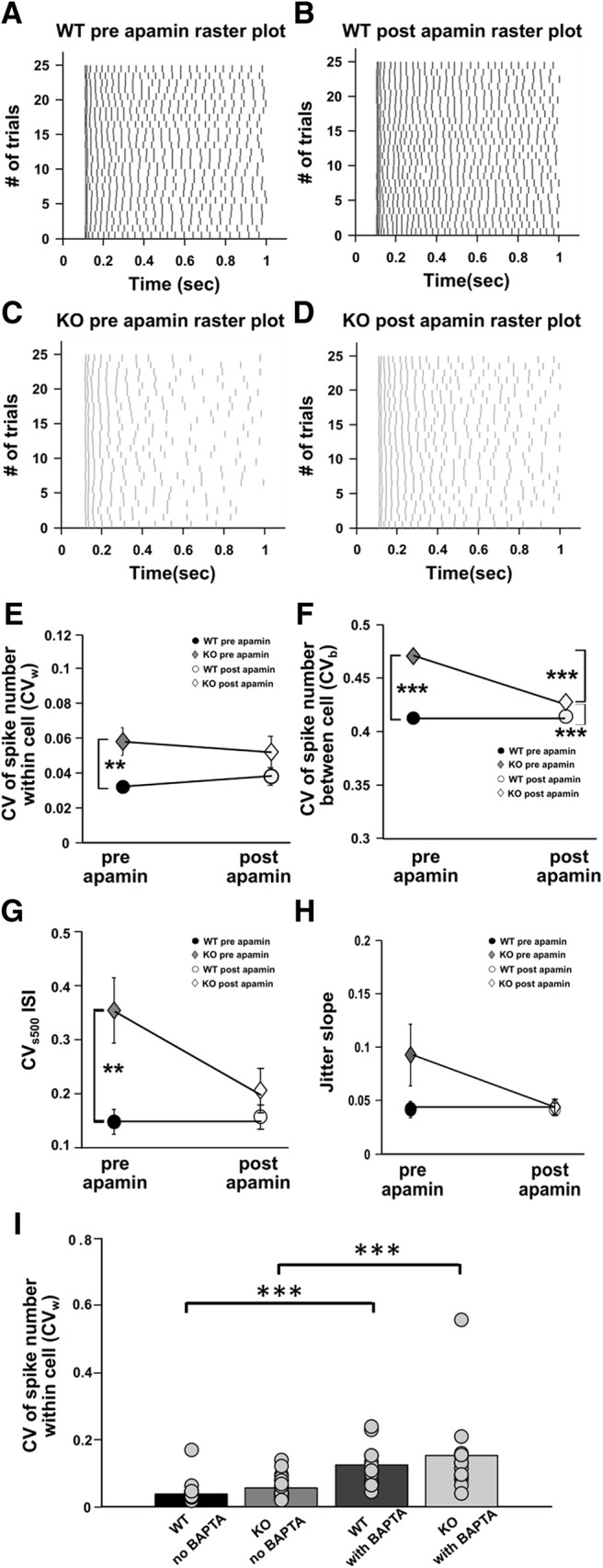
Partial rescue of within-cell variability (CV*_w_*), between-cell variability (CV*_b_*), and spike imprecision (CV*_s_*_500_) in KO neurons on apamin perfusion. ***A***, Representative raster plot showing spiking for step depolarization protocol for WT, pre-apamin perfusion. ***B***, Representative raster plot showing spiking for step depolarization protocol for WT, post-apamin perfusion. Same cell as in ***A***. ***C***, Representative raster plot showing spiking for step depolarization protocol for KO, pre-apamin perfusion. ***D***, Representative raster plot showing spiking for step depolarization protocol for KO, post-apamin perfusion. Same cell as in ***C***. ***E***, Change in within-cell variability parameter (CV*_w_*) for pre-apamin (filled black circle) and post-apamin perfusion in WT (*n* = 13 cells, empty black circle) and in KO (*n* = 13 cells, filled gray diamond for pre-apamin perfusion and empty gray diamond for post-apamin perfusion). There is a partial rescue in CV*_w_* parameter post-apamin perfusion for KO cells versus WT cells (*F*_(1,48)_ = 0.96 for effect of apamin; *F*_(1,48)_ = 0.004 for WT and KO differences; two-way ANOVA, error bars represent the SEM). ***F***, Plot showing change in between-cell variability parameter (CV*_b_*) for pre-apamin perfusion and post-apamin perfusion WT (*n* = 13 cells, filled black circle for pre-apamin perfusion and empty black circle for post-apamin perfusion) and for pre-apamin perfusion and post-apamin perfusion KO (*n* = 13 cells, filled gray diamond for pre-apamin perfusion and empty gray diamond for post-apamin perfusion). There is a partial rescue in CV*_b_* parameter for KO cells versus WT cells (*F*_(1,96)_ = 157.5; *p* < 0.001 for WT and KO differences; *F*_(1,96)_ = 53.1; *p* < 0.001 for effect of apamin on CV*_b_*_;_ two-way ANOVA, error bars represent the SEM). ***G***, Plot for CV*_s_*_500_ for ISI, a measure of spike precision for pre-apamin perfusion and post-apamin perfusion WT (*n* = 13 cells, filled black circle for pre-apamin perfusion and empty black circle for post-apamin perfusion) and for pre-apamin perfusion and post-apamin perfusion KO (*n* = 13 cells, filled gray diamonds for pre apamin and empty gray diamonds for post apamin). There is a partial rescue of CV*_s_*_500_ parameter on apamin perfusion (*F*_(1,49)_ = 9.63; *p* = 0.003 for WT and KO differences; *F*_(1,49)_ = 2.87; *p* = 0.09 for effect of apamin; two-way ANOVA, error bars represent the SEM). ***H***, Plot for jitter slope, a measure of spike precision for pre-apamin perfusion and post-apamin perfusion WT (*n* = 13 cells, filled black circle for pre-apamin perfusion and empty black circle for post-apamin perfusion) and for pre-apamin perfusion and post-apamin perfusion KO (*n* = 13 cells, filled gray diamonds for pre apamin and empty gray diamonds for post apamin). The jitter slope analysis did not report a significant difference in spike imprecision for WT versus KO cells (*F*_(1,41)_ = 3.63; *p* = 0.06 for WT and KO differences; *F*_(1,41)_ = 2.12; *p* = 0.15 for effect of apamin; two-way ANOVA, error bars represent the SEM). ***I***, Bar plot showing effect of BAPTA on within-cell variability (CV*_w_*) for WT (*n* = 30 cells without BAPTA and *n* = 12 cells with BAPTA) and KO (*n* = 29 cells without BAPTA and *n* = 12 cells with BAPTA). Chelating Ca^2+^ using BAPTA leads to a significant increase in spike variability within cell (CV*_w_*) for both WT and KO cells (*F*_(1,81)_ = 2.7; *p* = 0.1 for differences between WT and KO; *F*_(1,81)_ = 40.5 *p* < 0.001 for the effect of BAPTA; two-way ANOVA with replication, error bars represent the SEM). ***p < 0.001, **p < 0.01.

Having shown that elevated and variable SK currents play a role in cellular firing variability, we then asked whether blockage of the SK current might rescue the phenotype of increased variability. We observed that WT and KO populations responded differently on blocking SK currents using apamin. In WT cells, the mean of within-cell variability (CV*_w_*) between trials increased (CV*_w_* for WT cells pre-apamin perfusion, 0.03 ± 0.002, *n* = 13 cells; CV*_w_* for WT cells post-apamin perfusion, 0.04 ± 0.005, *n* = 13 cells; Fig. 8*A*,*B*,*E*). However, for KO cells, the mean of within-cell variability (CV*_w_*) between trials decreased (CV*_w_* for KO cells pre-apamin perfusion, 0.06 ± 0.008, *n* = 13 cells; CV*_w_* for KO cells post-apamin perfusion, 0.05 ± 0.009, *n* = 13 cells; Fig. 8*C*,*D*,*E*). Apamin treatment had no significant effect on within-cell variability (CV*_w_*) for either WT or KO. However, it leads to a partial rescue such that post-apamin perfusion KO CV*_w_* values were not significantly different from pre-apamin perfusion WT CV*_w_* values. A complete rescue by apamin would have resulted in a significant reduction of the CV*_w_* of KO cells (which did not happen) as well as a convergence of variability between KO and WT cells (which did) (*F*_(1,49)_ = 0, *p* = 0.96 for the effect of apamin perfusion; *F*_(1,48)_ = 8.74, *p* = 0.005 for WT and KO differences; two-way ANOVA, *t* test with Bonferroni correction; Fig. 8*E*). We performed similar analysis for between-cell (CV*_b_*) variability. Apamin perfusion led to a partial rescue in the CV*_b_* parameter as well, such that there was a significant reduction in the CV*_b_* of KO cells on apamin perfusion, but no convergence of variability between WT and KO cells (CV*_b_* for WT cells pre-apamin perfusion, 0.413 ± 0.002; CV*_b_* for WT cells post-apamin perfusion, 0.414 ± 0.003; CV*_b_* for KO cells pre-apamin perfusion, 0.47 ± 0.003; CV*_b_* for KO cells post-apamin perfusion, 0.43 ± 0.002; *F*_(1,96)_ = 157.5, *p* < 0.001 for WT and KO differences; *F*_(1,96)_ = 53.1; *p* < 0.001 for effect of apamin on CV*_b_*_;_ two-way ANOVA, *t* test with Bonferroni correction; Fig. 8*F*). CV*_s_*_500_ also showed a partial rescue with apamin perfusion (CV*_s_*_500_WT_ pre-apamin perfusion, 0.15 ± 0.02; CV*_s_*_500_WT_ post-apamin perfusion, 0.16 ± 0.02; CV*_s_*_500_KO_ pre-apamin perfusion, 0.35 ± 0.06; CV*_s_*_500_KO_ post-apamin perfusion, 0.21 ± 0.04; *F*_(1,49)_ = 9.63; *p* = 0.003 for WT and KO differences; *F*_(1,49)_ = 2.87, *p* = 0.09 for effect of apamin; two-way ANOVA, *t* test with Bonferroni correction; Fig. 8*G*). However, the jitter slope analysis was not able to recapitulate the rescue effects (jitter slope for WT cells pre-apamin perfusion, 0.04 ± 0.007; jitter slope for WT cells post-apamin perfusion, 0.04 ± 0.006; jitter slope for KO cells pre-apamin perfusion, 0.09 ± 0.03; jitter slope for KO cells post-apamin perfusion, 0.04 ± 0.007; *F*_(1,41)_ = 2.1; *p* = 0.15 for effect of apamin; *F*_(1,41)_ = 3.6; *p* = 0.06 for WT and KO differences; two-way ANOVA, *t* test with Bonferroni correction (Fig. 8*H*). Overall, we find that the blockage of SK currents by apamin partially rescues the variability phenotypes, which is consistent with our interpretation that the elevation in SK currents has a role in variability.

### Blocking calcium has no rescue affect

From the above observations, blocking SK currents led to only a partial rescue in the phenotype of increased imprecision and unreliability for spikes in KO cells. We therefore asked whether Ca^2+^, as an upstream modulator of SK, could also contribute to a partial rescue. To do this, we used BAPTA to chelate intracellular Ca^2+^. We added BAPTA (10 mm) into the intracellular recording pipette and assessed spike precision 10 min after patching. We found that chelating calcium using BAPTA led to a further increase in spike variability instead of rescue. Spike reliability within cell was measured (CV*_w_*) in these experiments, and it was found that spikes became significantly more variable post-BAPTA administration than pre-BAPTA for both WT and FXS cells (CV*_w_* for WT cells without BAPTA administration, 0.038 ± 0.005, *n* = 30 cells; CV*_w_* for WT cells with BAPTA administration, 0.12 ± 0.02, *n* = 12 cells; *p* < 0.001; CV*_w_* for KO cells without BAPTA administration, 0.057 ± 0.006, *n* = 29 cells; CV*_w_* for KO cells with BAPTA administration, 0.16 ± 0.04; *F*_(1,81)_ = 40.5, *p* < 0.001 for the effect of BAPTA; two-way ANOVA, *t* test with Bonferroni correction; Fig. 8*I*). Since Ca^2+^ has a large number of ion channels as other targets, we concluded that it was not a good target for the rescue of the variability phenotype.

## Discussion

**Figure 9.**
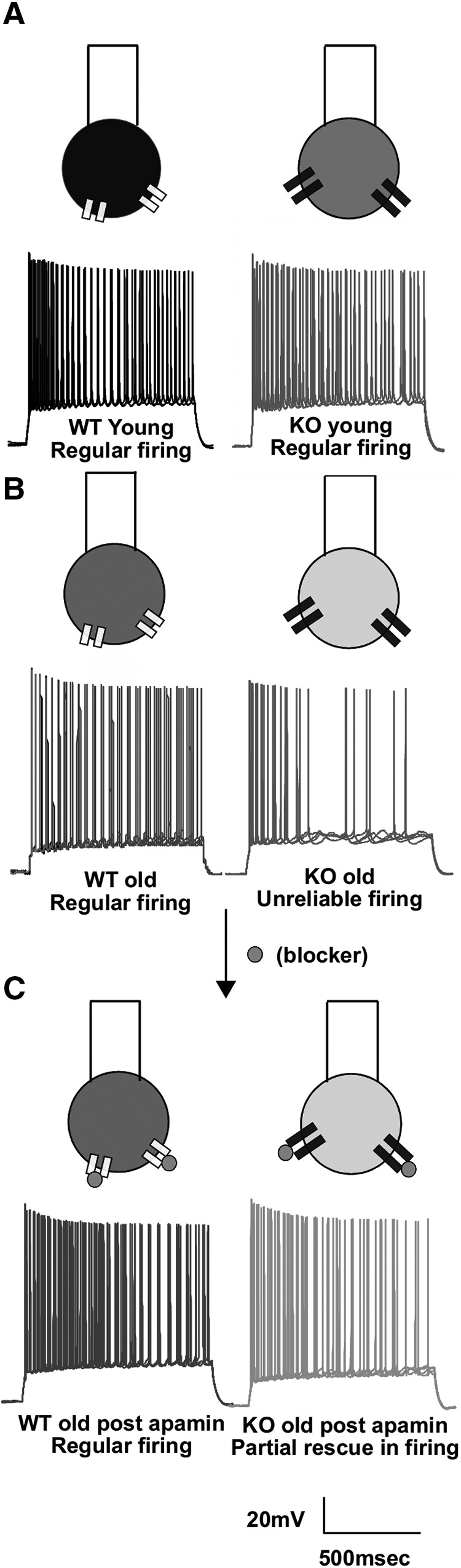
Schematic summary plot indicating a developmental increase in spiking variability and the role of SK channels. All spiking traces are actual raw data and show superimposed traces for four trials in WT (black) and KO (gray), respectively. ***A***, Schematic of neurons, showing younger WT (black) and younger KO (gray). In young animals, we observe precise and reliable spiking across multiple trials. Calibration: 20 mV, 500 ms. ***B***, Schematic of older WT and older KO neurons. KO neurons have larger SK currents, which are represented by larger icons for SK channels. Noticeably, KO spikes are more variable and less precise, especially in the later part of the trial. ***C***, Apamin (shown as small gray circles) is used to block SK channels present on soma. On apamin perfusion, spiking in WT (black) becomes more variable, whereas spiking in KO (light gray) increases in frequency, leading to increased spike precision and reliability.

**Figure 10.**
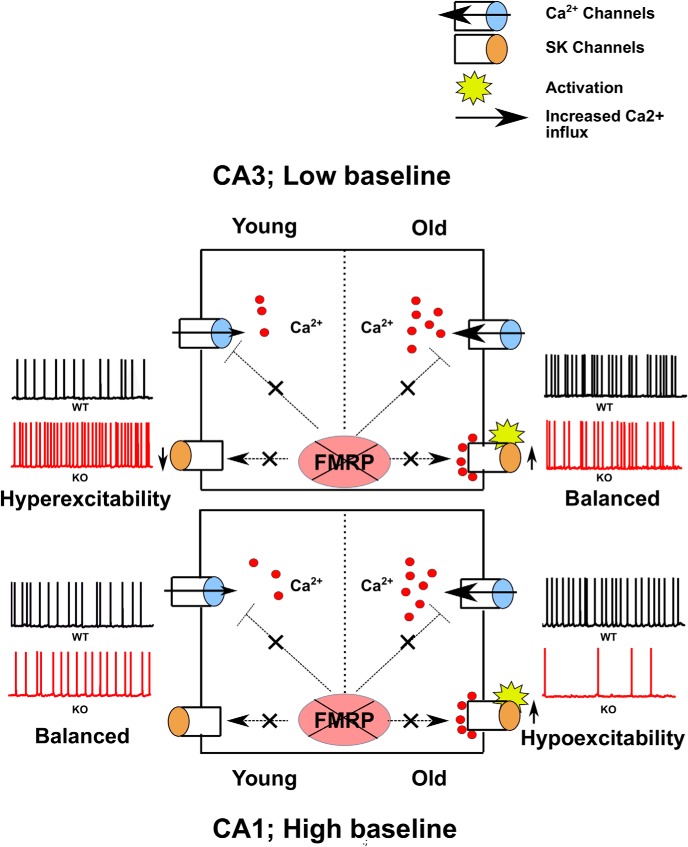
Schematic of a model explaining differential effect of FMRP KO in CA3 and CA1. ***A***, In young CA3 cells, the loss of FMRP leads to reduced activation of SK channels, which in turn gives neuronal hyperexcitability. However, as the animal matures, there is an increase in calcium influx in these cells. This compensates for this reduced SK activity seen in young CA3 cells leading to a nonsignificant change in excitability for KO cells compared with WT cells. ***B***, In young CA1 cells, both WT and KO cells start with elevated excitability as a result of which no significant differences can be seen between WT and KO. However, as the animal matures, the increase in Ca^2+^ influx brings excitability down for both WT and KO. This decrease in excitability is significantly greater for KO animals as many Ca^2+^ channels are known to be translationally regulated by FMRP. Under the absence of FMRP, these channels are known to be elevated significantly, making KO neurons hypoexcitable compared with WT neurons.

We have used a mouse model of autism, the fragile X knock-out mouse, to investigate cellular correlates of autism in hippocampal CA1 pyramidal neurons. We find that the reliability of spiking is impaired in FXS adult animals. We observed an increase in spiking variability both within a cell over multiple trials and between cells in matching trials, in FXS animals. The variability appeared between 6 and 8 weeks of age, consistent with current understanding of autism as a neurodevelopmental disorder. We observed that the increased spiking variability was accompanied by reduced spiking and elevated mAHP currents. We further dissected out the channel contributions to the elevated mAHP and found that SK currents were elevated in adult FXS neurons. However, levels of SK channels were not different between WT and KO mice. To test the hypothesis that elevated SK currents are responsible for unreliable and imprecise spiking in FXS neurons, we used the specific SK blocker apamin and obtained a partial rescue of the phenotype of increased spike variability. In addition to this, we discovered a differential effect of FMRP KO in different hippocampal regions. In agreement with previous published results, we found that there is an increase in excitability in CA3 region of hippocampus in juvenile animals. At the same age, CA1 cells do not show any difference in excitability between WT and KO animals. As the animal becomes older, we observe that the increased excitability seen in CA3 goes away, and for CA1 cells in this age group there is an observation of reduced excitability. This is the first study that has shown that FMRP KO can lead to very different outcomes in different regions of the same brain structure, further highlighting the complexity of the disorder (Figs. 9, 10).

### Lowered spike reliability and spike imprecision at cellular intrinsic level in FXS KO hippocampal cells

Our study demonstrates that there is a cell-intrinsic contribution to spiking variability in a mouse model of autism and implicates increased currents from SK channels as part of the mechanism. This finding takes significance with the failure of mGluR antagonists and blockers to significantly alleviate FXS symptoms in human patients. These findings point to the likelihood that the mGluR theory (Erickson et al., 2017) may be incomplete. Specifically, our finding of within-cell variability and between-cell variability in FXS cells shows that cells in the mutant animals may form a heterogeneous population of intrinsically variable neurons, the combined effects of which may lead to network-level incoherence.

This variability in neuronal responses over multiple trials may have implications for multiple behaviors. For example, in learning, the stimulus typically needs to be repeated over multiple trials. If the response of a cell at its intrinsic level as well as between cells is variable, the precision of input spiking as well as output activity would be reduced. This would adversely impact learning rules such as STDP and associativity (Markram et al., 1997; Bi and Poo, 1998). Further, elevated SK currents will have a direct impact on the recorded spontaneous EPSPs at the soma (Ngo-Anh et al., 2005).

### SK channel currents elevation may underlie previously observed age-dependent changes in KO cellular physiology

FXS has many clinical and experimental attributes of a neurodevelopmental syndrome (Nimchinsky et al., 2001; Cruz-Martín et al., 2010; Meredith et al., 2012). An age-dependent decline in learning has been shown in the *Drosophila* fragile X model (Choi et al., 2010). Theta burst stimulation (TBS) LTP has been showed to produced exaggerated LTP in anterior piriform cortex for older KO mice (12–18 months of age) but not in younger mice (<6 months of age; Larson, 2005). Similar results were seen in prefrontal cortex where TBS produced a significant impairment in LTP for 12-month-old mice but not in 2-month-old animals (Martin et al., 2016). Even in humans, it has been shown that the males who have a premutation (have CGG repeats <200) predominantly demonstrated fragile X-associated tremor/ataxia syndrome among patients who were >50 years old (Cornish et al., 2008). At the molecular level, an age-dependent change has also been shown in protein levels in FXS from postnatal day 17 (P17) mice to adults (Tang et al., 2015).

We observed a similar age-dependent change in the spike variability phenotype, which was absent in young KO animals, but manifested in older (>6 weeks old) mice (Fig. 3*D*). Age-dependent changes in SK channels have been previously reported such that the levels of SK channels are elevated in older animals compared with younger ones (Blank et al., 2003). This complements our finding that the AHP difference between WT and KO animals arose in older animals (Fig. 5) and that the SK channel underlay the difference in the older animals. There is also evidence of age-related changes in Ca^2+^ levels and Ca^2+^ uptake mechanisms, such that basal Ca^2+^ levels are elevated in aging brains (Das and Ghosh, 1996; Kirischuk et al., 1996; Foster and Norris, 1997). Such altered Ca^2+^ levels would also have a direct impact on the functionality of SK channels. We were not able to directly investigate this possible upstream effect due to the very diverse outcomes of modulating calcium signaling (Fig. 8*I*).

### SK channels may be an FMRP target with multiple physiologic and behavioral outcomes

FMRP targets multiple downstream pathways. It not only is a translational regulator but its N terminus is known to interact with multiple other proteins affecting their functioning (Brown et al., 2010; Deng et al., 2013). Thus, mutations in FMRP affect multiple aspects of cellular functioning. Previous RNA sequencing results have not shown that SK channels are elevated or affected in FXS (Brown et al., 2001; Darnell et al., 2011). However, the functioning of SK currents might be subject to other indirect effects, such as changes in calcium channel distribution, or in their functioning (Meredith et al., 2007; Ferron et al., 2014). Alterations in SK channels have substantial implications for multiple other phenotypes at the cellular and behavioral level. Previous studies have found that FXS KOs have impaired LTP in CA1 hippocampus (Lauterborn et al., 2007; Lee et al., 2011b; Tian et al., 2017). We suggest that this impairment might be partially due to elevated SK currents, which are known to shunt EPSPs and also to increase NMDA Mg^2+^ block (Ngo-Anh et al., 2005). Hence, our observation of elevated SK currents is consistent with the reported increase in LTP threshold in FXS (Lee et al., 2011b). These effects on LTP may have knock-on effects on memory phenotypes in FXS models. For example, a mild impairment in learning due to a change in platform location in the Morris water maze test has been found in FXS models (D'Hooge et al., 1997; Dobkin et al., 2000). SK currents have been shown to be reduced after learning, and this reduction leads to decreased variability in spiking events (Sourdet et al., 2003).

### Comparison with previously published studies

Multiple previous studies have found hyperexcitability in hippocampus in FXS model mice (Deng and Klyachko, 2016; Luque et al., 2017). We do not find a strong significant difference between KO and WT f–I curves (Fig. 3*I–L*). However, the previously published studies were conducted in the FVB strain of mice and the present study was performed in BL/6 mice. Strain-dependent differences in FXS have previously been found (Paradee et al., 1999; Dobkin et al., 2000; Lee et al., 2011b; Routh et al., 2013). However, Brager et al. (2012) found a lowering of input resistance for KO CA1 cells indicative of reduced excitability. Telias et al. (2015) have found a phenotype similar to ours in human hippocampal culture cells. In their study, they found that FXS cells were incapable of producing multiple spikes on sustained depolarization at multiple holding potentials, similar to what we observe in this study. It is interesting to note that most excitability analyses and f–I curves use 500 ms current steps, whereas in our study a difference arises after 600 ms and leads to a reduction in excitability in the KO (Figs. 1*B*, 3*G*). This suggests that a nuanced interpretation of such excitability studies is desirable, as the responses may have complex temporal features not reducible to a single measure of excitability. Another important point of difference is the age of the animals. Previous published studies have been performed on relatively young animals (<P30) compared with the present study, where adult animals (6–8 weeks of age) have also been used for the experiments. Our own results (Fig. 4*B*,*C*) show that there is a substantial change in spiking variability as well as AHPs as the animal matures. Other studies have also reported an age-dependent effect of the expression of plasticity phenotypes in FXS mice (Larson, 2005; Martin et al., 2016). Fragile X syndrome is known to be a developmental disease where symptoms and many other biochemical and other pathways are known to undergo changes with the age of the animal. Hence, it may be important to factor in age dependence and possibly use a wider range of ages to re-examine some of the conclusions that have been made from FXS models.

Our study is also the first to examine the effect of variability and spiking asynchrony, which again we find to be an age-dependent effect. We postulate that variability in the firing of individual cells, which previous studies have not explicitly examined, may be a further complicating factor in analyzing baseline changes arising due to the FMRP mutation.

### FMRP KO has different physiologic outcomes in different regions of the hippocampus brain regions

In the present study, we make the important and unexpected finding that FMRP KO has differential cell-physiologic effects on different subsections of the same brain region. In an attempt to replicate previous results, we found that there is hyperexcitability in pyramidal neurons of the CA3 region in juvenile mice, (as previously reported by Deng et al., 2019) but there was no significant difference in spiking in the CA1 region at the same age. Further, the phenotype reverses for CA1 cells in adult FMRP KO animals such that pyramidal neurons have reduced excitability, whereas for CA3 regions the hyperexcitability goes away. We suggest that these differential effects arise from the balance of effects among the direct effects of FMRP on SK channels, the indirect effects of FMRP on SK via calcium, and the effects of age, again potentially via calcium.

#### Direct effects of FMRP on SK

Deng et al. (2019) have shown that the absence of FMRP leads to reduced activity of SK channels. They have shown that a physical interaction between FMRP and SK leads to the activation of SK channels. This suggests that FMRP is a direct activator of SK channels. In the case of mutant animals where FMRP is absent, there is a reduction in SK currents leading to hyperexcitability, which has been observed both in the present and previous study (Fig. 10). We see this effect in CA3 cells for KO animals in the younger age group.

#### Indirect effects on SK via calcium

SK channels are dependent on intracellular calcium for their activation. Calcium channels are known targets of FMRP for transcription regulators (Brown et al., 2001; Darnell et al., 2011). Calcium influx via L-type Ca^2+^ channels are known to be elevated in neural progenitor cells (Danesi et al., 2018). Cortical culture neurons have been shown to have significantly elevated basal calcium levels in FXS neurons (Castagnola et al., 2018), and there is increased calcium influx in CA3 cells (Deng et al., 2011, 2013). In reference to human patients, multiple point mutations in Ca^2+^ genes are associated with autism (Contractor et al., 2015; Pinggera et al., 2015; Nguyen et al., 2018). There are multiple other studies in other neuron types that show increased intracellular calcium in autism models (Laumonnier et al., 2006; Krey and Dolmetsch, 2007; Tessier and Broadie, 2011; Pinggera et al., 2015; Limpitikul et al., 2016). These studies are in agreement with our observations in the present study where we see increased SK currents in CA1 cells, which is expected if the intracellular calcium levels are elevated in FXS neurons (Fig. 10). This increased calcium influx can explain the reduced excitability phenotypes seen in older CA3 and CA1 cells.

##### Effects of age on calcium

Calcium binding proteins and intracellular calcium dynamics are known to undergo development-dependent alterations in FXS neurons (Tessier and Broadie, 2011). Calcium levels and Ca^2+^ uptake mechanisms are also known to undergo changes with aging in animals (Das and Ghosh, 1996; Kirischuk et al., 1996; Foster and Norris, 1997). Though there are limited studies available in reference to a change in calcium channel levels with age in autism models, we hypothesize that a change in calcium dynamics is consistent with the fact that autism is a neurodevelopment disorder (Fig. 10). We see this effect in both CA3 and CA1 regions for KO animals, where in CA3 the increased excitability seen in younger animals levels off as the animal becomes adult. On the other hand, because of the increase in calcium, CA1 cells in which excitability was balanced in the young age group goes in the direction of hypoexcitability in older age groups.

Based on these findings, we propose a schematic model to explain these differences (Fig. 10). In the young age group for KO CA3 cells, the absence of FMRP leads to hyperexcitability, as explained above. As the animal becomes older, this effect goes away due to increased calcium influx with age, leading to SK activation and balancing out hyperexcitability. On the other hand, for young KO CA1 cells, both WT and KO start with maximum increased excitability where it is not significantly different. As the animal becomes older, increased calcium influx moves KO cells toward hypoexcitability.

### Defects in single-cell physiology may lead to network and population asynchrony

Multiple studies have shown that variability or lack of coordination in spiking exists in FXS at the network level (Arbab et al., 2018b; Talbot et al., 2018). In these tetrode-based studies, variability has been shown at the level of cellular firing patterns in the *in vivo* network; hence, it is difficult to separate cell-intrinsic properties from synaptic and network phenomena. In our study, we use the CV*_b_* parameter to gain an understanding of the correlation between cells at a population level. Using this analysis, we have tried to estimate the variability that exists between individual neuron responses when actually neurons were recorded one at a time. In contrast to tetrode recordings or field response recordings, where the recorded response is a result of multiple neurons and hence is a population response, CV*_b_* remains an intrinsic response of neurons depending on its active channels. CV*_b_* is an indirect readout of network correlations and should not be inferred as giving actual network correlation information. Clear mechanisms are lacking for this reported asynchrony. However, prior researchers have postulated that synaptic mechanisms may be responsible for the observed asynchrony (Talbot et al., 2018). Our study provides an alternative hypothesis for the mechanism, namely, that the effect is cell intrinsic and is in part due to altered kinetics of SK channels. Other studies provide evidence in favor of a role for SK channels in mediating precision and synchronous firing. For example, Deister et al. (2009) showed that SK channels were responsible for mediating precision in autonomous firing in globus pallidus neurons. Schultheiss et al. (2010) showed that dendritic SK channels also control synchronization. Another study by Kleiman-Weiner et al. (2009) has shown that SK channels play a role in mediating thalamocortical oscillations. A study by Combe et al. (2018) shows that SK channels contribute to phase locking for the spiking of CA1 cells of the hippocampus to slow gamma frequency inputs from Schaffer collaterals. Thus, the alterations in SK channel kinetics in FXS mutants as established by our study might have substantial impacts on phase coding and synchronization of hippocampal cell populations.

### A theory of FXS pathophysiology

An alternate theory to mGluR theory in explanation of FXS pathophysiology is the discoordination theory. The idea behind this theory is that many of the cognitive impairments seen in FXS are due to altered coordination in the spiking of cells in networks, which might be due either to an imbalance in excitation-inhibition in the networks (El Idrissi et al., 2005; Heulens et al., 2012) or to an intrinsic incoherent tendency of the neurons (Brock et al., 2002; Fenton, 2016; Radwan et al., 2016). Though this theory is preliminary, it may offer a promising alternative point of view from the traditional mGluR theory. Some studies have shown that intrinsic changes in cells during FXS affect their excitatory/inhibitory balance (Zhang et al., 2014; Lee et al., 2017) and that modulation of these channels leads to the rescue of the FXS phenotype. This present study has probed the dysfunction of a specific ion channel, SK, which was shown to affect the spiking variability at the single-cell level. It is an addition to the increasing list of ion channels that are known to be affected in FXS and may have an important effect on FXS etiology.

## Synthesis

Reviewing Editor: Alfonso Araque, University of Minnesota Decisions are customarily a result of the Reviewing Editor and the peer reviewers coming together and discussing their recommendations until a consensus is reached. When revisions are invited, a fact-based synthesis statement explaining their decision and outlining what is needed to prepare a revision will be listed below. The following reviewer(s) agreed to reveal their identity: Sonia Gasparini, Conrado Bosman.

The reviewers have found merit and interest on the findings reported. I concur with the reviewers' comments expressing the interest of the study. However, they also expressed some concerns about several issues that need to be addressed and clarified. I also consider pertinent the concerns expressed.

I am looking forward to receive a revised version of the manuscript after the concerns expressed by the reviewers have been addressed.

Best regards,

Alfonso Araque

Specific comments of the reviewers:

Reviewer 1.

This manuscript examines the neuronal properties of WT and FMRP-KO mice from two age groups, finding that excitability is decreased, and spiking variability is increased, only in the older KO in the CA1 region. This effect is due to an increase of the activity of SK channels, and can be partially reversed by the perfusion of apamin.

The results are potentially interesting, but I have some concerns with how some of the results were obtained and are presented, and the conclusions drawn from them as detailed above.

Comments on statistics:

The statistical analyses are not clear. It appears to me that two-way ANOVA should be run in most cases when there is an independent variable and two factors (for example age and genotype), and post-hoc tests, with correction, when significance is achieved. Maybe this is what has been done already; if so, the table at the end of the results should be adjusted accordingly.

Major points:

Since data for juvenile CA3 neurons are shown, and excitability increase in the KOs, I think that it would be very interesting to see what happens to CA3 neurons in older KO mice.

The statistical analyses are not clear. It appears to me that two-way ANOVA should be run in most cases when there is an independent variable and two factors (for example age and genotype), and post-hoc tests, with correction, when significance is achieved. Maybe this is what has been done already; if so, the table at the end of the results should be adjusted accordingly.

The various panels in the figures should be labeled (i.e. WT or KO, ages etc.). The lack of labels makes it difficult to understand what is represented in each of them

How was the resting Vm controlled in the current clamp experiments? There appear to be some fluctuations in the initial level in some of the traces (see for example Fig. 2B), and these differences, if real, could have a significant effect on neuronal firing.

Results and Methods: it is unclear how the jitter was calculated. What does spike time (or timing of the spike) means? the explanation in Fig. 1D is not helpful; it would be clearer if an inset showed the value used. On the same subject, what do the symbols in Fig 1I represent (one cell or all cells), and how was the slope calculated? Fitting the diamonds with a straight line does not appear appropriate.

Results page 7 lines 129-131. This sentence is vague. How many cells were reliable and how many were not? Reporting at least the proportion would be helpful

Figure 1F and others, and Methods lines 927-929: The calculation of CVb is unclear. How were the individual traces matched?

Fig. 2E and F: I don't think the experimental points should be linked by a line, since they are separate types of experiments. In addition, the symbol in Fig. 2F should be made bigger for clarity. Moreover, the error bars appear to be missing in Fig. 2F

Fig. 3F: What age groups are the data from? How was the fitting determined?

Fig. 3I and J: The protocol used to obtain the data should be reported. From the example in Figure 3I it appears that the f-I curves are shifted to the right with respect to WT, but this piece of information appears lost. Rather than just comparing the slope of the curves, it might be more informative to show average f-I plots, or to find a way to show this shift.

Fig. 6 C-F. The plots show multiple comparison of the same four groups. It would make more sense in my opinion to show only one plot with all the groups, and the statistical comparison on the side.

Results page 33 lines 573-575: this statement is unclear, since CWw for KO neurons actually decreased in the presence of apamin

Discussion lines 706-707: how can the authors say that overexpression of SK channels occurs, when that doesn't seem to be the case from their data (Fig. 7)?

Discussion line 822-828. The authors should clearly identify the mechanisms occurring in CA3 that result in hyperexcitability and CA1, where the result is decreased excitability. Rather than direct or indirect effect of FMRP, maybe a distinction by hippocampal region might be better?

Minor points:

Fig 4 legend (line343-344 and 351-352). What does “above traces...” mean in this context? Were the traces shown obtained at different Vm? Also, it would be useful to label CA3 and CA1 experiments in the figure itself

In the methods it should be clearly specified which experiments were conducted in voltage-clamp and which ones in current-clamp

Methods lines 962-963: Sag is a voltage deflection, not a current

Methods line 973: XE 991 is not a toxin

Methods lines 1003-1014: this paragraph is confusing and should be rewritten. Also, it is said in one place that Kolmogorov-Smirnov was used to test normality, whereas here it is said that normality was checked by plotting the data.

Reviewer 2.

This manuscript studies the cellular mechanisms underlying spike variability in a mouse model of Fragile X disease. The authors hypothesized that the cellular mechanisms leading to spike variability in the hippocampus of FMRP knockout (KO) mice are related to the functioning of potassium channels, which have shown to have effects on spike precision. The authors used patch clamp recording techniques in slices of KO and WT mice. They observed that the spiking activity of hippocampal neurons from KO mice is indeed more unreliable and imprecise than controls. This effect appears to emerge in adult mice (8 weeks) but not in juvenile mice (6 weeks of age), it seems to be region-specific, i.e. CA3 but not in CA1. Mechanistically, it seems to be related to a decrease in spike number and elevated after membrane hyperpolarization due to elevated potassium currents. Importantly, this phenotype was partially recovered using apamine, a specific SK blocker. The authors concluded that cellular mechanisms such as the conductance of potassium channels may be involved in the generation of the spike variability in FMRP KO model.

In general, the hypotheses and questions of this manuscript are timely and interesting, the experiments are well described, and the results and interpretations are, in general, sound. That being said, I think the manuscript requires substantial revisions aiming to improve the text readiness, and the scope of the interpretation of the results.

My major concern is about the use of the Coefficient of Variation between neurons (CV), which attempt to measure the spike variability across different neurons recorded non-simultaneously. The rationale of the CVb measurement is unclear, especially if the idea was to compare CVb differences between KO and WT, with those results about spike precision across cells obtained in extracellular recordings using tetrodes. In those studies, cells are recorded simultaneously, and some idea of the network properties can be inferred. Although the stimulation protocols are uniform and mimic some of the conditions observed in vivo, it is important to highlight the fact that the CVb does not necessarily reflect network interactions. I think the authors should clarify this point in the discussion section.

Also, an extended description of the CVb method, pointing to some examples of the literature, would help non-specialized readers. The methodological explanation is given between L107 and L110, and L111 and below sounds redundant and ambiguous.

Figure 2 (E and F) related to these analyses is unsatisfactory. There is no point to show connected lines between protocols because there are independent between them. While the rationale for the analysis is the same, the layout of the two panels is different. For instance, the former shows error bars, but the latter does not. (Is Ran 10 also significant?).

Finally, the authors mention during the discussion that these results are somewhat different from those expected under the Glutamate Theory. This idea could be expanded including the concept of inhibitory and excitatory disbalance as a fundamental physiopathological mechanism in FXS (cf. Fenton, A. A. (2015). Excitation-inhibition discoordination in rodent models of mental disorders. Biological Psychiatry, 77(12), 1079-1088. http://doi.org/10.1016/j.biopsych.2015.03.013) and how elevated potassium currents can contribute to this misbalance.

Minor:

While I am not a native English speaker myself, I noticed several sentences that are not well constructed. For instance:

L83: “...if there were...”

L191: “...phenotypic differences which manifest for the...”

L331:“there was no difference”

L364 “guided by the above”

L373 “In brief,” it shouldn't be used in the context of that sentence

L526 “the levels of SK2 protein was not...” should be replaced by were.

L694-L695 Are some words missed? “Spiking rates (?)”

And probably others... I suggest asking for a manuscript editorial checking.

2) Related to the previous point, I noticed some unnecessary redundancy in the description of results (e.g., L244 to L256, compared with L237 and L240). Please condense the text in a clearer description

2) Check the layout of the figures:

1) Figures require to show the color convention in every panel. Otherwise, it is difficult to follow what pertains to WT and what is referred to KO

1) panels seem to be not well aligned (Fig 1I and 1J, Fig 3G and H).

2) The traces in Figure5A and B (the zooming lines are not well aligned to the squares)

3) X-labels are missed in Fig 4B and D

## Author Response

Manuscript Instructions

- The sex of the species studied is not mentioned in the materials and methods section. Please mention “male”, “female”, “x males and x females”, “of either sex”.

Author Response

---------------

The authors would thank for pointing this out. Corrections have been made as shown below

C57BL/6 strain of Fmr1tmCgr male mice were used for the experiments.

- Materials and Methods should follow the Introduction.

Author Response

---------------

The edits have been made.

Specific comments of the reviewers:

===================================

# Reviewer 1.

-------------

This manuscript examines the neuronal properties of WT and FMRP-KO mice from two age groups, finding that excitability is decreased, and spiking variability is increased, only in the older KO in the CA1 region. This effect is due to an increase of the activity of SK channels, and can be partially reversed by the perfusion of apamin.

The results are potentially interesting, but I have some concerns with how some of the results were obtained and are presented, and the conclusions drawn from them as detailed above.

Comments on statistics:

The statistical analyses are not clear. It appears to me that two-way ANOVA should be run in most cases when there is an independent variable and two factors (for example age and genotype), and post-hoc tests, with correction, when significance is achieved. Maybe this is what has been done already; if so, the table at the end of the results should be adjusted accordingly.

Author Response

---------------

We would like to thank the reviewer for suggesting these improvements to the statistics. We have implemented the two-way ANOVA in several places, as suggested, and performed post-hoc tests as appropriate. These changes have been made in Fig 3E, 3F, 3G, 3J and Fig 5C, 5D. Necessary changes have been made in the table at the end of the results section.

Major points:

Since data for juvenile CA3 neurons are shown, and excitability increase in the KOs, I think that it would be very interesting to see what happens to CA3 neurons in older KO mice.

Author Response

---------------

Upon reviewer?s suggestion, CA3 experiments were done for the older animals as well. Results from the paper are copied below. These findings are also summarized as a schematic in the final figure as figure 10.

Upon seeing this differential effect of FMRP KO in different regions of hippocampus, we were interested to see if this might be a function of age. Therefore, we recorded similar protocols from CA3 and CA1 of older animals. We found that the hyper-excitability seen in younger CA3 cells, goes away, leading to no significant difference in spiking activity in these cells for WT vs KO (# of spikes in 60 s for -48mV holding potential for WT 313.6 ñ 83.8, n=10 cells; # of spikes in 60 s for -48mV holding potential for KO 181.1 ñ 54.9, n=10 cells; F (1, 75) = 1.16, p=0.28 for difference between WT and KO, two-way ANOVA) (Figure 4E, 4F). In agreement with our previous results as shown in Fig 3, we observed reduced excitability in CA1 KO cells as compared to WT (# of spikes in 60 s for -50mV holding potential for WT 135.6 ñ 35.2, n=8 cells; # of spikes in 60 s for -50mV holding potential for KO 54 ñ 20.4, n=8 cells; F (1, 59) = 7.42, p=0.009 for difference between WT and KO, two-way ANOVA) (Figure 4G, 4H). Using th ese experiments, we were able to replicate previous published results of Deng et. al. 2019. In addition, we found that there were contrasting and age-dependent effects of FMRP KO on the spiking properties of CA1 and CA3 pyramidal neurons.

The statistical analyses are not clear. It appears to me that two-way ANOVA should be run in most cases when there is an independent variable and two factors (for example age and genotype), and post-hoc tests, with correction, when significance is achieved. Maybe this is what has been done already; if so, the table at the end of the results should be adjusted accordingly.

Author Response

---------------

Done, as indicated above.

The various panels in the figures should be labeled (i.e. WT or KO, ages etc.). The lack of labels makes it difficult to understand what is represented in each of them

Author Response

---------------

The suggested changes have been made in the figure labelling.

How was the resting Vm controlled in the current clamp experiments? There appear to be some fluctuations in the initial level in some of the traces (see for example Fig. 2B), and these differences, if real, could have a significant effect on neuronal firing.

Author Response

---------------

To address this point, explanation was added in the figure text as shown below

The resting Vm was controlled by maintaining constant current injection into the cell while recording. Cells were only accepted if the fluctuations in their potential while recording were ñ 2.5mV.

Results and Methods: it is unclear how the jitter was calculated. What does spike time (or timing of the spike) means? the explanation in Fig. 1D is not helpful; it would be clearer if an inset showed the value used.

Author Response

---------------

The authors would like to thank the reviewer for pointing out the ambiguity in the jitter analysis method. We have added a detailed explanation of the analysis method in methods section as shown below

The jitter slope analysis was taken from (Bacci and Huguenard, 2006). Jitter was defined as åti, which is the standard deviation of spike timing for spike number i, compared over all trials for a given cell. In this manner the vector åt was obtained by considering all the spike numbers. Note that not all trials had the same number of spikes, so the final few spike numbers may have had fewer samples in åt.(Extended figure 1-1).

The Jitter slope was obtained as a regression fit for the vector åt , with the x axis defined by the corresponding vector for mean timings æt. This slope was calculated for spikes falling in the last 400 ms of the current pulse since this is where the SK channel is activated. As a pre hoc criterion, cells were required to have a minimum of two spike time points in order to be selected for this calculation. Note that this criterion excluded several cells which had strong suppression of firing towards the end of the trial, presumably due to SK channel activation. Thus this measure was compromised by being insensitive to precisely those cells with the largest effects in the KO.(Extended data 1-1)

On the same subject, what do the symbols in Fig 1I represent (one cell or all cells), and how was the slope calculated? Fitting the diamonds with a straight line does not appear appropriate.

Author Response

---------------

The authors would like to thank the reviewer for pointing this out. We agree that fitting a straight line to the entire jitter distribution was not the correct analysis. Thus, we analyzed the jitter only in the last 400ms of the trial. This part of the trial shows peak activity of SK currents and hence is most relevant to our analysis. However, due to the requirement that a cell should have a minimum of two spikes for a regression fit, several KO cells got rejected. As a result, this analysis was unable to show spike precision aspects of those KO cells which had probably the strongest effect of elevated SK channel activity.

The updated jitter Slope analysis has been included in the paper as Extended data 1-1, 3-1 and 8-1. Detailed explanation for the analysis is added in the methods section, excerpted above.

As a preferred method, we examined Coefficient of variation of Inter-Spike Interval (CVs of ISI) to analyze spike timing differences In Fig1I, 1J, 3F and 8J. The analysis method is described below.

Spike times were found for all the spikes in all trials. The Inter Spike Interval (ISI) was computed for the first two spikes and for spikes that bracketed a time of 500ms after the start of the step input protocol. For each cell m we obtained the Coefficient of variation, CVs0_m and CVs500_m for the first ISI and the ISI at 500 ms, respectively:

CVs0_m = å0m/æ0m Equation 3.

Where m is the cell index, åm is the standard deviation of ISI across trials within cell m, and æm is the mean ISI across all the trials for cell m. We calculated CVs500_m in a similar manner.

By repeating this calculation over all cells, we obtained the following four vectors: CVs0_WT, CVs500_WT, CVs0_KO, and CVs500_KO. The distributions in these four vectors were compared using a two-way ANOVA.

Results page 7 lines 129-131. This sentence is vague. How many cells were reliable and how many were not? Reporting at least the proportion would be helpful

Author Response

---------------

To address this point, explanation was added in the figure text as shown below

Based on this analysis (Fig 1G and 1H) we found that 51.7% of KO cells had CVw values which did not overlap with those of WT cells.

Figure 1F and others, and Methods lines 927-929: The calculation of CVb is unclear. How were the individual traces matched?

Author Response

---------------

Thank you for pointing out the lack of clarity. We have provided a more detailed explanation in materials and methods:

The analysis of CVb method was derived from a paper which reports experiments on of network synchrony (Talbot et al., 2018). In contrast to our CVw analysis where variability was assessed across trials within cell, in CVb we measure across trials between cells. For computing between-cell spiking variability (CVb), spike numbers on corresponding trial numbers were compared between different cells. Specifically, we constructed a vector for each cell whose entries were the number of spikes for successive trials. These vectors were compared across different cells, within the same genotype and used to find CVb. The following formula was used for the computation:

(CVb)i = åi/æi Equation 2.

Where i is the trial index, åi is the standard deviation over trial i between cells, and æi is the mean number of spikes on trial i, between all cells. The CVb vectors were computed for WT and KO populations respectively (Figure 1F) and compared using Wilcoxon statistics.

Fig. 2E and F: I don't think the experimental points should be linked by a line, since they are separate types of experiments. In addition, the symbol in Fig. 2F should be made bigger for clarity. Moreover, the error bars appear to be missing in Fig. 2F

Author Response

---------------

To improve clarity and understanding, the line plots have been replaced with bar plot. Also error bars were not missing in 2F. They were just smaller than the symbols used. However, in the new plot, individual data points are plotted superimposed as scatter plot on a bar plot, for clarity.

Fig. 3F: What age groups are the data from? How was the fitting determined?

Author Response

---------------

The age groups are mentioned below each individual bar in the bar plot. However, the information about which age group was used for fitting data was missing in Fig 3H, in the legends. Corrections have been made.

Fig. 3I and J: The protocol used to obtain the data should be reported. From the example in Figure 3I it appears that the f-I curves are shifted to the right with respect to WT, but this piece of information appears lost. Rather than just comparing the slope of the curves, it might be more informative to show average f-I plots, or to find a way to show this shift.

Author Response

---------------

We thank the reviewer for pointing out the lack of clarity. An extra section in methods has been added to explain the measurement and plotting of f-I curves, as shown below. Also, extra panels with average f-I curves, as suggested by reviewer has also been added as Fig 3K and 3L

The measurement of f-I curves was done in current clamp. For plotting f-I curves, each cell was given input currents from -100pA to 400pA at its resting membrane potential. The mean ñ SEM of the number of spikes elicited for each input current was plotted on the Y axis, and the corresponding input current on the X axis.

Fig. 6 C-F. The plots show multiple comparison of the same four groups. It would make more sense in my opinion to show only one plot with all the groups, and the statistical comparison on the side.

Author Response

---------------

We thank the reviewer for the suggestion. We tried this. However, the condensed figure was cluttered and difficult to interpret, so we reverted to the original expanded form for clarity. The condensed figure is attached here to show why we chose to expand it out.

In the above figure, the line with diamond markers is KO old. Line with circles markers (WT old), plus markers (KO young) and star markers (WT young) all nearly overlap with each other..

Results page 33 lines 573-575: this statement is unclear, since CWw for KO neurons actually decreased in the presence of apamin

Author Response

---------------

We have clarified this in the results section as shown below

A complete rescue by apamin would have resulted in a significant reduction of the CVw of KO cells (which did not happen) as well as a convergence of variability between KO and WT (which did)

Discussion lines 706-707: how can the authors say that overexpression of SK channels occurs, when that doesn't seem to be the case from their data (Fig. 7)?

Author Response

---------------

The author would thank the reviewer for pointing out the mistake. Corrections have been made as show below

Our study demonstrates that there is a cell-intrinsic contribution to spiking variability in a mouse model of autism, and implicates increased currents from SK channels as part of the mechanism.

Discussion line 822-828. The authors should clearly identify the mechanisms occurring in CA3 that result in hyperexcitability and CA1, where the result is decreased excitability. Rather than direct or indirect effect of FMRP, maybe a distinction by hippocampal region might be better?

Author Response

---------------

We would like to thank the reviewer for pointing out lack of clarity on this point. We have expanded the explanation for this point into a distinct sub section in the discussion titled ?FMRP KO has different physiological outcomes in different regions of the hippocampus brain regions?. Further, we have added fig 10 to explain the distinction of FMRP KO in different hippocampal regions.

Minor points:

Fig 4 legend (line343-344 and 351-352). What does “above traces...” mean in this context? Were the traces shown obtained at different Vm? Also, it would be useful to label CA3 and CA1 experiments in the figure itself

Author Response

---------------

Thanks for pointing this out.

?Above traces? refer to the representative traces shown in the figure. This has been replaced with the clarification that these are representative traces in the figure legends. These traces are the representative traces from one of the holding voltage used to induce spiking in the neurons (Fig 4A, C, E, G). Details have been added in the legend as shown below

A) Representative traces showing increased excitability between WT (black) and KO (grey) CA3 cells in younger animals. Traces are from the recording where the cells are held at -48mV. Scale bar 20mV, 1s.

.

Also suggested labelling has been done to improve clarity.

In the methods it should be clearly specified which experiments were conducted in voltage-clamp and which ones in current-clamp

Author Response

---------------

Changes have been made to clarify which experiments were done in which mode. Text from the methods section is shown below

All spike variability and precision experiments were done in current clamp mode.

The measurement of f-I curves was done in current clamp.

AHPs were measured by holding the cell at -70mV in Current Clamp mode.

For measuring Ih sag produced in voltage change, cells were held at -70mV and given a step hyperpolarization of 250pA for 500msec, in current clamp mode.

TTX (0.5 æM) was added in the bath while measuring M currents in voltage-clamp

TTX (0.5 æM) was added in the bath while measuring SK currents in voltage-clamp.

Methods lines 962-963: Sag is a voltage deflection, not a current

Author Response

---------------

Changes have been made as shown below

Ih channel activity was recorded using a hyperpolarization pulse. The voltage sag produced in response to the pulse, was quantified according to a method adapted from Brager and Johnston, 2012 (Methods, Fig 5E).

Methods line 973: XE 991 is not a toxin

Author Response

---------------

Changes have been made as shown below

M current specific blocker XE991 was used to obtain the post blocker trace.

Methods lines 1003-1014: this paragraph is confusing and should be rewritten. Also, it is said in one place that Kolmogorov-Smirnov was used to test normality, whereas here it is said that normality was checked by plotting the data.

Author Response

---------------

Changes have been made as shown below

Statistical tests were done after checking the normality of the data. We plotted the data to check for normality. Even if the data looked normal, Wilcoxon rank sum test was used for most analysis for more stringent results.

# Reviewer 2.

----------------

This manuscript studies the cellular mechanisms underlying spike variability in a mouse model of Fragile X disease. The authors hypothesized that the cellular mechanisms leading to spike variability in the hippocampus of FMRP knockout (KO) mice are related to the functioning of potassium channels, which have shown to have effects on spike precision. The authors used patch clamp recording techniques in slices of KO and WT mice. They observed that the spiking activity of hippocampal neurons from KO mice is indeed more unreliable and imprecise than controls. This effect appears to emerge in adult mice (8 weeks) but not in juvenile mice (6 weeks of age), it seems to be region-specific, i.e. CA3 but not in CA1. Mechanistically, it seems to be related to a decrease in spike number and elevated after membrane hyperpolarization due to elevated potassium currents. Importantly, this phenotype was partially recovered using apamine, a specific SK blocker. The authors concluded that cellular mechanisms such as the conductance of potassium channels may be involved in the generation of the spike variability in FMRP KO model.

In general, the hypotheses and questions of this manuscript are timely and interesting, the experiments are well described, and the results and interpretations are, in general, sound.

Author Response

---------------

We thank the reviewer for their positive comments.

That being said, I think the manuscript requires substantial revisions aiming to improve the text readiness, and the scope of the interpretation of the results.

My major concern is about the use of the Coefficient of Variation between neurons (CV), which attempt to measure the spike variability across different neurons recorded non-simultaneously. The rationale of the CVb measurement is unclear, especially if the idea was to compare CVb differences between KO and WT, with those results about spike precision across cells obtained in extracellular recordings using tetrodes. In those studies, cells are recorded simultaneously, and some idea of the network properties can be inferred. Although the stimulation protocols are uniform and mimic some of the conditions observed in vivo, it is important to highlight the fact that the CVb does not necessarily reflect network interactions. I think the authors should clarify this point in the discussion section.

Author Response

---------------

The authors would thank the reviewers for pointing this out and relevant corrections have been added in the text as added below.

In our study, we use the CVb parameter to gain an understanding about the correlation between cells at a population level. Using this analysis we have tried to estimate the variability which exists between individual neuron responses when actually neurons were recorded one at a time. In contrast to tetrode recordings or field response recordings, where the recorded response is a resultant of multiple neurons and hence is a population response, CVb remains an intrinsic response of neurons depending on its active channels. CVb is an indirect readout of network correlations and should not be inferred as giving actual network correlation information.

Also, an extended description of the CVb method, pointing to some examples of the literature, would help non-specialized readers. The methodological explanation is given between L107 and L110, and L111 and below sounds redundant and ambiguous.

Author Response

---------------

The analysis of CVb method was derived from a paper as shown below. Also the description of CVb analysis method was explained in detail in methods section as shown below

The analysis of CVb method was derived from a paper which reports experiments on of network synchrony (Talbot et al., 2018). In contrast to our CVw analysis where variability was assessed across trials within cell, in CVb we measure across trials between cells. For computing between-cell spiking variability (CVb), spike numbers on corresponding trial numbers were compared between different cells. Specifically, we constructed a vector for each cell whose entries were the number of spikes for successive trials. These vectors were compared across different cells, within the same genotype and used to find CVb. The following formula was used for the computation:

(CVb)i = åi/æi Equation 2.

Where i is the trial index, åi is the standard deviation over trial i between cells, and æi is the mean number of spikes on trial i, between all cells. The CVb vectors were computed for WT and KO populations respectively (Figure 1F) and compared using Wilcoxon statistics.

Figure 2 (E and F) related to these analyses is unsatisfactory. There is no point to show connected lines between protocols because there are independent between them. While the rationale for the analysis is the same, the layout of the two panels is different. For instance, the former shows error bars, but the latter does not. (Is Ran 10 also significant?).

Author Response

---------------

To improve clarity and understanding, the line plots have been replaced with bar plot. Also error bars were not missing in 2F. They were just smaller than the symbols used. However, in the new plot, individual data points are plotted superimposed as scatter plot on a bar plot, for clarity. However, significance showing stars were missing in Ran 10 by mistake. That has been corrected.

Finally, the authors mention during the discussion that these results are somewhat different from those expected under the Glutamate Theory. This idea could be expanded including the concept of inhibitory and excitatory disbalance as a fundamental physiopathological mechanism in FXS (cf. Fenton, A. A. (2015). Excitation-inhibition discoordination in rodent models of mental disorders. Biological Psychiatry, 77(12), 1079-1088. http://doi.org/10.1016/j.biopsych.2015.03.013) and how elevated potassium currents can contribute to this misbalance.

Author Response

---------------

The authors would thank the reviewers for the suggestion. An extra section has been added in the discussion section as shown below.

A theory of FXS pathophysiology

An alternate theory to mGLUR theory in explanation of FXS pathophysiology is the ?discoordination? theory. The idea behind this theory, is that many of the cognitive impairments seen in FXS are due to altered coordination in spiking of cells in networks, which might be either due to an imbalance in excitation-inhibition in the networks (El Idrissi et al., 2005; Heulens et al., 2012) or due to an intrinsic incoherent tendency of the neurons (Brock et al., 2002; Fenton, 2015; Radwan et al., 2016). Though this theory is preliminary, it may offer a promising alternative point of view from the tranditional mGLUR theory. Some studies have shown that intrinsic changes in cells during FXS effect their E/I balance (Zhang et al., 2014; Lee et al., 2017) and modulation of these channels lead to rescue of FXS phenotype This present study has probed dysfunction of a specific ion channel, SK which was shown to effect the spiking variability at single cell level. It is an addition to the increasing list of ion channels which are known to be affected in FXS and may have an important effect on FXS etiology.

Minor:

While I am not a native English speaker myself, I noticed several sentences that are not well constructed. For instance:

L83: “...if there were...”

L191: “...phenotypic differences which manifest for the...”

L331:“there was no difference”

L364 “guided by the above”

L373 “In brief,” it shouldn't be used in the context of that sentence

L526 “the levels of SK2 protein was not...” should be replaced by were.

L694-L695 Are some words missed? “Spiking rates (?)”

And probably others... I suggest asking for a manuscript editorial checking.

2) Related to the previous point, I noticed some unnecessary redundancy in the description of results (e.g., L244 to L256, compared with L237 and L240). Please condense the text in a clearer description

Author Response

---------------

The authors would like to thank the reviewer for pointing these mistakes. We have fixed them. The text has been condensed for clarity. It is shown below

Using post hoc tests we concluded that for KO cells there is a worsening of phenotype for both variability in spiking number (CVw) across multiple trials within a cell and in spike timing precision (CVs500 of ISI) across multiple trials within a cell (Fig 3E and 3F, detailed statistics shown in table 1).

Check the layout of the figures:

1) Figures require to show the color convention in every panel. Otherwise, it is difficult to follow what pertains to WT and what is referred to KO

Author Response

---------------

Extra labelling has been done in each figure for better clarity.

1) panels seem to be not well aligned (Fig 1I and 1J, Fig 3G and H).

2) The traces in Figure5A and B (the zooming lines are not well aligned to the squares)

Author Response

---------------

The alignments have been made.

3) X-labels are missed in Fig 4B and D

Author Response

---------------

Figure has been edited to rectify the mistake.

## References

[B1] Arbab T, Battaglia FP, Pennartz CMA, Bosman CA (2018a) Abnormal hippocampal theta and gamma hypersynchrony produces network and spike timing disturbances in the Fmr1-KO mouse model of fragile X syndrome. Neurobiol Dis 114:65–73. 10.1016/j.nbd.2018.02.011 29486296

[B2] Arbab T, Pennartz CMA, Battaglia FP (2018b) Impaired hippocampal representation of place in the Fmr1-knockout mouse model of fragile X syndrome. Sci Rep 8:8889. 10.1038/s41598-018-26853-z 29892074 PMC5995880

[B3] Bacci A, Huguenard JR (2006) Enhancement of spike-timing precision by autaptic transmission in neocortical inhibitory interneurons. Neuron 49:119–130. 10.1016/j.neuron.2005.12.014 16387644

[B4] Bi G, Poo M (1998) Synaptic modifications in cultured hippocampal neurons: dependence on spike timing, synaptic strength, and postsynaptic cell type. J Neurosci 18:10464–10472. 9852584 10.1523/JNEUROSCI.18-24-10464.1998 PMC6793365

[B5] Blank T, Nijholt I, Kye MJ, Radulovic J, Spiess J (2003) Small-conductance, Ca2+-activated K+channel SK3 generates age-related memory and LTP deficits. Nat Neurosci 6:911–912. 10.1038/nn1101 12883553

[B6] Brager DH, Akhavan AR, Johnston D (2012) Impaired dendritic expression and plasticity of h-channels in the fmr1-/y mouse model of fragile X syndrome. Cell Rep 1:225–33. 10.1016/j.celrep.2012.02.002 22662315 PMC3363364

[B7] Brock JON, Brown CC, Boucher J (2002) The temporal binding deficit hypothesis of autism. Dev Psychopathol 14:209–224. 12030688 10.1017/s0954579402002018

[B8] Brown MR, Kronengold J, Gazula VR, Chen Y, Strumbos JG, Sigworth FJ, Navaratnam D, Kaczmarek LK (2010) Fragile X mental retardation protein controls gating of the sodium-activated potassium channel Slack. Nat Neurosci 13:819–21. 10.1038/nn.2563 20512134 PMC2893252

[B9] Brown V, Jin P, Ceman S, Darnell JC, Donnell WTO, Tenenbaum SA, JX, Feng Y, Wilkinson KD, Keene JD, Darnell RB, Warren ST (2001) Microarray identification of FMRP-associated brain mRNAs and altered mRNA translational profiles in fragile X syndrome. Cell 107:477–487. 10.1016/S0092-8674(01)00568-2 11719188

[B10] Carandini M (2004) Amplification of trial-to-trial response variability by neurons in visual cortex. PLoS Biol 2:E264. 10.1371/journal.pbio.0020264 15328535 PMC509408

[B11] Carlen PL (1997) Reversible inhibition of I K, I AHP, I h and I Ca currents by internally applied gluconate in rat hippocampal pyramidal neurones. Pflugers Arch 433:343–350. 10.1007/s004240050286 9064651

[B12] Castagnola S, Delhaye S, Folci A, Paquet A, Brau F, Duprat F, Jarjat M, Grossi M, Béal M (2018) New insights into the role of Cav2 protein family in calcium flux deregulation in Fmr1-KO neurons. Front Mol Neurosci 11:1–13. 30319351 10.3389/fnmol.2018.00342 PMC6170614

[B13] Choi CH, McBride SMJ, Schoenfeld BP, Liebelt DA, Ferreiro D, Ferrick NJ, Hinchey P, Kollaros M, Rudominer RL, Terlizzi AM, Koenigsberg E, Wang Y, Sumida A, Nguyen HT, Bell AJ, McDonald TV, Jongens TA (2010) Age-dependent cognitive impairment in a Drosophila Fragile X model and its pharmacological rescue. Biogerontology 11:347–362. 10.1007/s10522-009-9259-6 20039205 PMC2866528

[B14] Combe CL, Canavier CC, Gasparini S (2018) Intrinsic mechanisms of frequency selectivity in the proximal dendrites of CA1 pyramidal neurons. J Neurosci 38:8110–8127. 30076213 10.1523/JNEUROSCI.0449-18.2018 PMC6146492

[B15] Contractor A, Klyachko VA, Portera-Cailliau C (2015) Altered neuronal and circuit excitability in fragile X syndrome. Neuron 87:699–715. 10.1016/j.neuron.2015.06.017 26291156 PMC4545495

[B16] Cornish KM, Li L, Kogan CS, Jacquemont S, Turk J, Dalton A, Hagerman RJ, Hagerman PJ (2008) Age-dependent cognitive changes in carriers of the fragile X syndrome. Cortex 44:628–636. 10.1016/j.cortex.2006.11.002 18472033 PMC11060834

[B17] Crook SM, Ermentrout GB, Bower JM (1998) Spike frequency adaptation affects the synchronization properties of networks of cortical oscillators. Neural Comput 10:837–854. 10.1162/089976698300017511 9573408

[B18] Cruz-Martín A, Crespo M, Portera-Cailliau C (2010) Delayed stabilization of dendritic spines in fragile X mice. J Neurosci 30:7793–7803. 10.1523/JNEUROSCI.0577-10.2010 20534828 PMC2903441

[B19] Cudmore RH, Fronzaroli-Molinieres L, Giraud P, Debanne D (2010) Spike-time precision and network synchrony are controlled by the homeostatic regulation of the D-type potassium current. J Neurosci 30:12885–12895. 10.1523/JNEUROSCI.0740-10.2010 20861392 PMC6633566

[B20] Danesi C, Achuta VS, Corcoran P, Peteri U, Turconi G (2018) Increased calcium influx through L-type calcium channels in human and mouse neural progenitors lacking fragile X mental retardation protein. Stem Cell Rep 11:1449–1461. 10.1016/j.stemcr.2018.11.003 30503263 PMC6294261

[B21] Darnell JC, Driesche SJ, Van Zhang C, Hung YK, Mele S, Fraser A, Stone CE, Chen EF, Fak C, Chi JJ, Licatalosi SW, Richter DD, Darnell JD, RB (2011) FMRP stalls ribosomal translocation on mRNAs linked to synaptic function and autism. Cell 146:247–261. 10.1016/j.cell.2011.06.013 21784246 PMC3232425

[B22] Das N, Ghosh S (1996) The effect of age on calcium dynamics in rat brain in vivo. Mech Ageing Dev 88:17–24. 10.1016/0047-6374(96)01713-7 8803919

[B23] Deister CA, Chan CS, Surmeier DJ, Wilson CJ (2009) Calcium-activated SK channels influence voltage-gated ion channels to determine the precision of firing in globus pallidus neurons. J Neurosci 29:8452–8461. 10.1523/JNEUROSCI.0576-09.2009 19571136 PMC3329865

[B24] Deng PY, Klyachko VA (2016) Increased persistent sodium current causes neuronal hyperexcitability in the entorhinal cortex of Fmr1 knockout mice. Cell Rep 16:3157–3166. 10.1016/j.celrep.2016.08.046 27653682 PMC5055130

[B25] Deng PY, Sojka D, Klyachko VA (2011) Abnormal presynaptic short-term plasticity and information processing in a mouse model of fragile X syndrome. J Neurosci 31:10971–10982. 10.1523/JNEUROSCI.2021-11.2011 21795546 PMC6623101

[B26] Deng PY, Rotman Z, Blundon JA, Cho Y, Cui J, Cavalli V, Zakharenko SS, Klyachko VA (2013) FMRP regulates neurotransmitter release and synaptic information transmission by modulating action potential duration via BK channels. Neuron 77:696–711. 10.1016/j.neuron.2012.12.018 23439122 PMC3584349

[B27] Deng PY, Carlin D, Oh YM, Myrick LK, Warren ST, Cavalli V, Klyachko VA (2019) Voltage-independent SK channel dysfunction causes neuronal hyperexcitability in the hippocampus of *Fmr1* knock-out mice. J Neurosci 39:28–43. 10.1523/JNEUROSCI.1593-18.2018 30389838 PMC6325266

[B28] D'Hooge R, Nagels G, Franck F, Bakker CE, Reyniers E, Storm K, Kooy RF, Oostra BA, Willems PJ, De Deyn PP (1997) Mildly impaired water maze performance in male Fmr1 knockout mice. Neuroscience 76:367–376. 10.1016/S0306-4522(96)00224-2 9015322

[B29] Dobkin C, Rabe A, Dumas R, El Idrissi A, Haubenstock H, Ted Brown W (2000) Fmr1 knockout mouse has a distinctive strain-specific learning impairment. Neuroscience 100:423–429. 10.1016/S0306-4522(00)00292-X 11008180

[B30] El Idrissi A, Ding XH, Scalia J, Trenkner E, Brown WT, Dobkin C (2005) Decreased GABA(A) receptor expression in the seizure-prone fragile X mouse. Neurosci Lett 377:141–146. 10.1016/j.neulet.2004.11.087 15755515

[B31] Erickson CA, Davenport MH, Schaefer TL, Wink LK, Pedapati EV, Sweeney JA, Fitzpatrick SE, Brown WT, Budimirovic D, Hagerman RJ, Hessl D, Kaufmann WE, Berry-Kravis E (2017) Fragile X targeted pharmacotherapy: lessons learned and future directions. J Neurodev Disord 9:1–14. 28616096 10.1186/s11689-017-9186-9 PMC5467059

[B32] Faisal AA, Selen LPJ, Wolpert DM (2008) Noise in the nervous system. Nat Rev Neurosci 9:292–303. 10.1038/nrn2258 18319728 PMC2631351

[B33] Fenton AA (2016) Excitation-inhibition discoordination in rodent models of mental disorders. Biol Psychiatry 77:1079–1088. 10.1016/j.biopsych.2015.03.013 PMC4444398 25895430

[B34] Ferron L, Nieto-Rostro M, Cassidy JS, Dolphin AC (2014) Fragile X mental retardation protein controls synaptic vesicle exocytosis by modulating N-type calcium channel density. Nat Commun 5:3628. 10.1038/ncomms4628 24709664 PMC3982139

[B35] Foster TC, Norris CM (1997) Age-associated changes in Ca(2+)-dependent processes: relation to hippocampal synaptic plasticity. Hippocampus 7:602–612. 10.1002/(SICI)1098-1063(1997)7:6<602::AID-HIPO3>3.0.CO;2-G 9443057

[B36] Fricker D, Miles R (2000) EPSP amplification and the precision of spike timing in hippocampal neurons. Neuron 28:559–569. 10.1016/s0896-6273(00)00133-1 11144364

[B37] Fujisawa S, Yamada MK, Nishiyama N, Matsuki N, Ikegaya Y (2004) BDNF boosts spike fidelity in chaotic neural oscillations. Biophys J 86:1820–1828. 10.1016/S0006-3495(04)74249-6 14990508 PMC1304016

[B38] Gabbiani F, Dewell R (2018) M-current regulates firing mode and spike reliability in a collision detecting neuron. J Neurophysiol 120:1753–1764. 10.1152/jn.00363.2018 30044671 PMC6230786

[B39] Gastrein P, Campanac É, Gasselin C, Cudmore RH, Bialowas A, Carlier E, Fronzaroli-Molinieres L, Ankri N, Debanne D (2011) The role of hyperpolarization-activated cationic current in spike-time precision and intrinsic resonance in cortical neurons in vitro. J Physiol 589:3753–3773. 10.1113/jphysiol.2011.209148 21624967 PMC3171884

[B40] Gittelman JX (2006) Kv1.1-containing channels are critical for temporal precision during spike initiation. J Neurophysiol 96:1203–1214. 10.1152/jn.00092.2005 16672305

[B41] Gonçalves JT, Anstey JE, Golshani P, Portera-Cailliau C (2013) Circuit level defects in the developing neocortex of fragile X mice. Nat Neurosci 16:903–909. 10.1038/nn.3415 23727819 PMC3695061

[B42] Gross C, Yao X, Pong DL, Jeromin A, Bassell GJ (2011) Fragile X mental retardation protein regulates protein expression and mRNA translation of the potassium channel Kv4.2. J Neurosci 31:5693–5698. 10.1523/JNEUROSCI.6661-10.2011 21490210 PMC3089949

[B43] Guan D, Higgs MH, Horton LR, Spain WJ, Foehring RC (2011) Contributions of Kv7-mediated potassium current to sub- and suprathreshold responses of rat layer II/III neocortical pyramidal neurons. J Neurophysiol 106:1722–1733. 10.1152/jn.00211.2011 21697446 PMC3191833

[B44] Han X, Boyden ES (2007) Multiple-color optical activation, silencing, and desynchronization of neural activity, with single-spike temporal resolution. PLoS One 2:e299. 10.1371/journal.pone.0000299 17375185 PMC1808431

[B45] Heulens I, D’Hulst C, Van Dam D, De Deyn PP, Kooy RF (2012) Pharmacological treatment of fragile X syndrome with GABAergic drugs in a knockout mouse model. Behav Brain Res 229:244–249. 10.1016/j.bbr.2012.01.031 22285772

[B46] Hönigsperger C, Marosi M, Murphy R, Storm JF (2015) Dorsoventral differences in Kv7/M-current and its impact on resonance, temporal summation and excitability in rat hippocampal pyramidal cells. J Physiol 593:1551–1580. 10.1113/jphysiol.2014.280826 25656084 PMC4386960

[B47] Hou GQ, Pan X, Liao CS, Wang SH, Li DF (2012) SK channels modulate the excitability and firing precision of projection neurons in the robust nucleus of the arcopallium in adult male zebra finches. Neurosci Bull 28:271–281. 10.1007/s12264-012-1241-7 22622827 PMC5560329

[B48] Kirischuk S, Voitenko N, Kostyuk P, Verkhratsky A (1996) Age-associated changes of cytoplasmic calcium homeostasis in cerebellar granule neurons in situ: investigation on thin cerebellar slices. Exp Gerontol 31:475–487. 10.1016/0531-5565(95)02070-5 9415105

[B49] Kleiman-Weiner M, Beenhakker MP, Segal WA, Huguenard JR (2009) Synergistic roles of GABA A receptors and SK channels in regulating thalamocortical oscillations. J Neurophysiol 102:203–213. 10.1152/jn.91158.2008 19386752 PMC2712277

[B50] Krey JF, Dolmetsch RE (2007) Molecular mechanisms of autism: a possible role for Ca2+ signaling. Curr Opin Neurobiol 17:112–119. 10.1016/j.conb.2007.01.010 17275285

[B51] Kwag J, Jang HJ, Kim M, Lee S (2014) M-type potassium conductance controls the emergence of neural phase codes: a combined experimental and neuron modelling study. J R Soc Interface 11:20140604. 25100320 10.1098/rsif.2014.0604 PMC4233740

[B52] Larson J (2005) Age-dependent and selective impairment of long-term potentiation in the anterior piriform cortex of mice lacking the fragile X mental retardation protein. J Neurosci 25:9460–9469. 10.1523/JNEUROSCI.2638-05.2005 16221856 PMC6725716

[B53] Laumonnier F, Roger S, Guérin P, Molinari F, M’rad R, Cahard D, Belhadj A, Halayem M, Persico AM, Elia M, Romano V, Holbert S, Andres C, Chaabouni H, Colleaux L, Constant J, Le Guennec JY, Briault S (2006) Association of a functional deficit of the BKCa channel, a synaptic regulator of neuronal excitability, with autism and mental retardation. Am J Psychiatry 163:1622–1629. 10.1176/ajp.2006.163.9.1622 16946189

[B54] Lauterborn JC, Rex CS, Krama E, Chen LY, Pandyarajan V, Lynch G, Gall CM (2007) Brain-derived neurotrophic factor rescues synaptic plasticity in a mouse model of fragile X syndrome. J Neurosci 27:10685–10694. 10.1523/JNEUROSCI.2624-07.2007 17913902 PMC6672822

[B55] Lee E, Lee J, Kim E (2017) Excitation/inhibition imbalance in animal models of autism spectrum disorders. Biol Psychiatry 81:838–847. 10.1016/j.biopsych.2016.05.011 27450033

[B56] Lee HY, Ge W-P, Huang W, He Y, Wang GX, Rowson-Baldwin A, Smith SJ, Jan YN, Jan LY (2011a) Bidirectional regulation of dendritic voltage-gated potassium channels by the fragile X mental retardation protein. Neuron 72:1091 10.1016/j.neuron.2011.11.012 PMC3433402 22099464

[B57] Lee HY, Ge W, Huang W, He Y, Wang GX, Smith SJ, Jan YN, Jan LY (2011b) Bidirectional regulation of dendritic voltage-gated potassium channels by the fragile X mental retardation protein. Neuron 72:630–642. 10.1016/j.neuron.2011.09.033 22099464 PMC3433402

[B58] Limpitikul WB, Dick IE, Ben-Johny M, Yue DT (2016) An autism-associated mutation in CaV1.3 channels has opposing effects on voltage- and Ca2+-dependent regulation. Sci Rep 6:1–13. 27255217 10.1038/srep27235 PMC4891671

[B59] Luque MA, Beltran-Matas P, Marin MC, Torres B, Herrero L (2017) Excitability is increased in hippocampal CA1 pyramidal cells of Fmr1 knockout mice. PLoS One 12:e0185067. 10.1371/journal.pone.0185067 28931075 PMC5607184

[B60] Madison DV, Lancaster B, Nicoll RA (1987) Voltage clamp analysis of cholinergic action in the hippocampus. J Neurosci 7:733–741. 3559710 10.1523/JNEUROSCI.07-03-00733.1987 PMC6569053

[B88] Mainen ZF, Sejnowski TJ (1995) Reliability of spike timing in neocortical neurons. Science 268:1503–1506. 7770778 10.1126/science.7770778

[B61] Markram H, Lübke J, Frotscher M, Sakmann B (1997) Regulation of synaptic efficacy by coincidence of postsynaptic APs and EPSPs. Science 275:213–215. 10.1126/science.275.5297.213 8985014

[B62] Martin HGS, Lassalle O, Brown JT, Manzoni OJ (2016) Age-dependent long-term potentiation deficits in the prefrontal cortex of the Fmr1 knockout mouse model of fragile X syndrome. Cereb Cortex 26:2084–2092. 10.1093/cercor/bhv031 25750254

[B63] Meredith RM, Dawitz J, Kramvis I (2012) Sensitive time-windows for susceptibility in neurodevelopmental disorders. Trends Neurosci 35:335–344. 10.1016/j.tins.2012.03.005 22542246

[B64] Meredith RM, Holmgren CD, Weidum M, Burnashev N (2007) Increased threshold for spike-timing-dependent plasticity is caused by unreliable calcium signaling in mice lacking fragile X gene Fmr1. Neuron 54:627–638. 10.1016/j.neuron.2007.04.028 17521574

[B65] Ngo-Anh TJ, Bloodgood BL, Lin M, Sabatini BL, Maylie J, Adelman JP (2005) SK channels and NMDA receptors form a Ca2+-mediated feedback loop in dendritic spines. Nat Neurosci 8:642–649. 10.1038/nn1449 15852011

[B66] Nguyen RL, Medvedeva YV, Ayyagari TE, Schmunk G, Jay J (2018) Intracellular calcium dysregulation in autism spectrum disorder: an analysis of converging organelle signaling pathways. Biochim Biophys Acta Mol Cell Res Res 1865:1718–1732. 10.1016/j.bbamcr.2018.08.003 30992134

[B67] Nigro MJ, Mateos-Aparicio P, Storm JF (2014) Expression and functional roles of Kv7/KCNQ/M-channels in rat medial entorhinal cortex layer II stellate cells. J Neurosci 34:6807–6812. 10.1523/JNEUROSCI.4153-13.2014 24828634 PMC6608108

[B68] Nimchinsky EA, Oberlander AM, Svoboda K (2001) Abnormal development of dendritic spines in FMR1 knock-out mice. J Neurosci 21:5139–5146. 11438589 10.1523/JNEUROSCI.21-14-05139.2001 PMC6762842

[B69] O’Donnell WT, Warren ST (2002) A decade of molecular studies of fragile X syndrome. Annu Rev Neurosci 25:315–338. 10.1146/annurev.neuro.25.112701.142909 12052912

[B70] Orbán G, Kiss T, Erdi P (2006) Intrinsic and synaptic mechanisms determining the timing of neuron population activity during hippocampal theta oscillation. J Neurophysiol 96:2889–2904. 10.1152/jn.01233.2005 16899632

[B71] Paradee W, Melikian HE, Rasmussen DL, Kenneson A, Conn PJ (1999) Fragile X mouse: strain effects of knockout phenotype and evidence suggesting deficient amygdala function. Neuroscience 94:185–192. 10.1016/s0306-4522(99)00285-7 10613508

[B72] Pfeuty B, Mato G, Golomb D, Hansel D (2003) Electrical synapses and synchrony: the role of intrinsic currents. J Neurosci 23:6280–6294. 12867513 10.1523/JNEUROSCI.23-15-06280.2003 PMC6740557

[B73] Pinggera A, Lieb A, Benedetti B, Lampert M, Monteleone S, Liedl KR, Tuluc P, Striessnig J (2015) CACNA1D de novo mutations in autism calcium channels. Biol Psychiatry 77:816–822. 10.1016/j.biopsych.2014.11.020 25620733 PMC4401440

[B74] Radwan B, Dvorak D, Fenton AA (2016) Impaired cognitive discrimination and discoordination of coupled theta-gamma oscillations in Fmr1 knockout mice. Neurobiol Dis 88:125–138. 10.1016/j.nbd.2016.01.003 26792400 PMC4758895

[B75] Routh BN, Johnston D, Brager DH (2013) Loss of functional A-type potassium channels in the dendrites of CA1 pyramidal neurons from a mouse model of fragile X syndrome. J Neurosci 33:19442–19450. 10.1523/JNEUROSCI.3256-13.2013 24336711 PMC3858620

[B76] Sah P, Clements JD (1999) Photolytic manipulation of [Ca2+]i reveals slow kinetics of potassium channels underlying the afterhyperpolarization in hipppocampal pyramidal neurons. J Neurosci 19:3657–3664. 10.1523/JNEUROSCI.19-10-03657.1999 10233997 PMC6782703

[B77] Schultheiss NW, Edgerton JR, Jaeger D (2010) Phase response curve analysis of a full morphological globus pallidus neuron model reveals distinct perisomatic and dendritic modes of synaptic integration. J Neurosci 30:2767–2782. 10.1523/JNEUROSCI.3959-09.2010 20164360 PMC2833015

[B89] Shah MM, Mistry M, Marsh SJ, Brown DA, Delmas P (2002) Molecular correlates of the M-current in cultured rat hippocampal neuronsn. J Physiol 544(Pt 1):29–37. 12356878 10.1113/jphysiol.2002.028571 PMC2290582

[B78] Sourdet V, Russier M, Daoudal G, Ankri N, Debanne D (2003) Long-term enhancement of neuronal excitability and temporal fidelity mediated by metabotropic glutamate receptor subtype 5. J Neurosci 23:10238–10248. 14614082 10.1523/JNEUROSCI.23-32-10238.2003 PMC6741009

[B79] Stevens CF, Zador AM (1998) Input synchrony and the irregular firing of cortical neurons. Nat Neurosci 1:210–217. 10.1038/659 10195145

[B90] Stocker M, Krause M, Pedarzani P (1999) An apamin-sensitive Ca2+-activated K+ current in hippocampal pyramidal neurons. Proc Natl Acad Sci U S A 96:4662–4667. 10200319 10.1073/pnas.96.8.4662 PMC16389

[B80] Strumbos JG, Brown MR, Kronengold J, Polley DB, Kaczmarek LK (2010) Fragile X mental retardation protein is required for rapid experience-dependent regulation of the potassium channel Kv3.1b. J Neurosci 30:10263–10271. 10.1523/JNEUROSCI.1125-10.2010 20685971 PMC3485078

[B81] Talbot ZN, Sparks FT, Dvorak D, Curran BM, Alarcon JM, Fenton AA (2018) Normal CA1 place fields but discoordinated network discharge in a Fmr1-null mouse model of fragile X syndrome. Neuron 684–697.e4. 10.1016/j.neuron.2017.12.043 29358017 PMC6066593

[B82] Tang B, Wang T, Wan H, Han L, Qin X, Zhang Y, Wang J, Yu C, Berton F, Francesconi W, Yates JR, Vanderklish PW, Liao L (2015) *Fmr1* deficiency promotes age-dependent alterations in the cortical synaptic proteome. Proc Natl Acad Sci 112:E4697–E4706. 10.1073/pnas.1502258112 26307763 PMC4553823

[B91] Telias M, Kuznitsov-Yanovsky L, Segal M, Ben-Yosef D (2015) Functional deficiencies in Fragile X neurons derived from human embryonic stem cells. J Neurosci 35:15295–15306. 26586818 10.1523/JNEUROSCI.0317-15.2015 PMC6605488

[B83] Tessier CR, Broadie K (2011) The fragile X mental retardation protein developmentally regulates the strength and fidelity of calcium signaling in Drosophila mushroom body neurons. Neurobiol Dis 41:147–159. 10.1016/j.nbd.2010.09.002 20843478 PMC2982942

[B84] Testa-Silva G, Loebel A, Giugliano M, De Kock CPJ, Mansvelder HD, Meredith RM (2012) Hyperconnectivity and slow synapses during early development of medial prefrontal cortex in a mouse model for mental retardation and autism. Cereb Cortex 22:1333–1342. 10.1093/cercor/bhr224 21856714 PMC3561643

[B85] Tian Y, Yang C, Shang S, Cai Y, Deng X, Zhang J, Shao F, Zhu D, Liu Y, Chen G, Liang J, Sun Q, Qiu Z, Zhang C (2017) Loss of FMRP impaired hippocampal long-term plasticity and spatial learning in rats. Front Mol Neurosci 10:1–14. 28894415 10.3389/fnmol.2017.00269 PMC5581399

[B86] Zhang Y, Bonnan A, Bony G, Ferezou I, Pietropaolo S, Ginger M, Sans N, Rossier J, Oostra B, LeMasson G, Frick A (2014) Dendritic channelopathies contribute to neocortical and sensory hyperexcitability in Fmr1−/y mice. Nat Neurosci 17:1701–1709. 25383903 10.1038/nn.3864

[B87] Zhu P, Li J, Zhang L, Liang Z, Tang B, Liao WP, Yi YH, Su T (2018) Development-related aberrations in Kv1.1 α-subunit exert disruptive effects on bioelectrical activities of neurons in a mouse model of fragile X syndrome. Prog Neuropsychopharmacol Biol Psychiatry 84:140–151. 10.1016/j.pnpbp.2018.02.011 29481897

